# Comparative metabolomic profiling of *Cupriavidus necator* B-4383 revealed production of cupriachelin siderophores, one with activity against *Cryptococcus neoformans*

**DOI:** 10.3389/fchem.2023.1256962

**Published:** 2023-08-24

**Authors:** Mohammed M. A. Ahmed, Siddarth K. Tripathi, Paul D. Boudreau

**Affiliations:** ^1^ Boudreau Lab, Department of BioMolecular Sciences, School of Pharmacy, University of Mississippi, Oxford, MS, United States; ^2^ Department of Pharmacognosy, Al-Azhar University, Cairo, Egypt; ^3^ National Center for Natural Products Research, School of Pharmacy, University of Mississippi, Oxford, MS, United States

**Keywords:** cupriachelin, siderophore, metabolomics (LC-MS), *Cupriavidus necator*, mass spectrometry

## Abstract

*Cupriavidus necator* H16 is known to be a rich source of linear lipopeptide siderophores when grown under iron-depleted conditions; prior literature termed these compounds cupriachelins. These small molecules bear *β*-hydroxyaspartate moieties that contribute to a photoreduction of iron when bound as ferric cupriachelin. Here, we present structural assignment of cupriachelins from *C. necator* B-4383 grown under iron limitation. The characterization of B-4383 cupriachelins is based on MS/MS fragmentation analysis, which was confirmed by 1D- and 2D-NMR for the most abundant analog (**1**). The cupriachelin congeners distinguish these two strains with differences in the preferred lipid tail; however, our rigorous metabolomic investigation also revealed minor analogs with changes in the peptide core, hinting at a potential mechanism by which these siderophores may reduce biologically unavailable ferric iron (**4–6**). Antifungal screening of the *C. necator* B-4383 supernatant extract and the isolated cupriachelin analog (**1**) revealed inhibitory activity against *Cryptococcus neoformans*, with IC_50_ values of 16.6 and 3.2 μg/mL, respectively. This antifungal activity could be explained by the critical role of the iron acquisition pathway in the growth and pathogenesis of the *C. neoformans* fungal pathogen.

## 1 Introduction

Siderophores, iron chelating small molecules, are produced by bacteria under iron-limiting conditions to maintain iron homeostasis, which is essential for growth and survival (Kramer et al., 2020). Many reports present non-conventional functions for bacterial siderophores, including the ability to work as metallophores for a variety of non-iron metals (Bobrov et al., 2014; Koh and Henderson, 2015; Robinson et al., 2018), sequester heavy metal toxins (Harrington et al., 2012; Bobrov et al., 2014), act as signaling molecules (Roux et al., 2009), and regulate oxidative stress (Adler et al., 2012). Exciting new applications of siderophores include use as antibiotics (Miethke and Marahiel, 2007; Frederick et al., 2009; Mislin and Schalk, 2014) or in bioremediation approaches for heavy metals (Johnston et al., 2013; Nancharaiah and Lens, 2015; Chen et al., 2016; Gadd and Pan, 2016; Li et al., 2016). In our laboratory, we are developing workflows to discover siderophores with new structures that might allow for these functionalities. In this work, the strain *C. necator* B-4383 was chosen as it is closely related to *C. necator* H16, which is known to produce lipopeptide siderophores (cupriachelins) (Kreutzer et al., 2012). These molecules bear the *β*-hydroxyaspartic acid (*β*-OH-Asp) functional group for Fe (III) coordination, which may also play a central role in complexation with non-iron heavy metals (Hardy and Butler, 2018). LCMS-based comparison of the small molecules excreted into a minimal iron-deficient medium by *C. necator* B-4383 *versus* the same medium supplemented with iron led to the identification of a series of presumed cupriachelin analogs. Isolation and structural characterization showed that the B-4383 cupriachelins bear differences in their lipid tail length (**1–3**), as previously reported for *C. necator* H16 (Kreutzer et al., 2012). However, our annotation of the MS/MS fragmentation spectra of these compounds also led to the identification of several minor analogs. An interesting set of analogs displayed a replacement of one of the *β*-hydroxyaspartic acids in the peptide backbone with a glycine moiety (**4–6**), which we hypothesize, based on a UV exposure experiment, are derived from the photoreduction of iron. Many other analogs show changes in the hydroxylation patterns or appear to be the products of dehydration and hydrolysis reactions, which may indicate that they are artifacts of the extraction process (**7–17**). Our research revealed a significant inhibitory activity against *C. neoformans*, but not *Candida albicans* or *Aspergillus fumigatus* by both the culture broth extract and a purified cupriachelin analog (**1**). *Cryptococcus neoformans* is a pathogenic fungus responsible for life-threatening meningoencephalitis in immunocompromised people such as AIDS patients or individuals receiving immunosuppressive therapy (Bicanic and Harrison, 2004). It has been previously reported that the iron acquisition pathway is critical for *C. neoformans* pathogenesis and its ability to express major virulence factors (Won et al., 2006; Jung et al., 2009; Saikia et al., 2014). We believe our work represents the first antifungal activity reported for cupriachelin siderophores.

## 2 Materials and methods

### 2.1 General experimental procedures

IR spectra were recorded on an Agilent Technologies Cary 630 FTIR spectrometer. Specific rotations were measured using a Rudolph AUTOPOL II Automatic Polarimeter. UV measurements were recorded on an Agilent Cary 5000 UV-Vis-NIR spectrophotometer. NMR spectra were measured on a Bruker Advance III HD 500 MHz spectrometer with a cryoprobe in deuterated water or deuterated water with 0.002% TMSP as an internal reference (Cambridge Isotopes). ^1^H NMR spectra were run at 500 MHz and ^13^C NMR spectra were produced at 125 MHz. HR-ESI-MS and MS/MS spectra were collected using an Agilent 6530C Q-TOF LC/MS system with an Agilent jet stream source and an Agilent 1,260 Infinity II pump stack. Sephadex LH-20 (25–100 μm; Cytiva) and RP-SPE C_18_ columns (100 mg, 1,000 mg, and 5 g; Thermo Scientific) were used for purification before HPLC. For the HPLC purification step, an Agilent 1,260 Infinity II HPLC system with a multiple wavelength detector and a fraction collector was used. A Sorvall Legend Mirco 21R centrifuge was used for centrifugation of cell pellets. Corning Lambda Plus single channel pipettors were used to handle small aliquots, with 0.2–2 μL, 2–20 μL, 20–200 μL, and 100–1,000 μL volume pipettors employed. A Fisherbrand Accumet XL150 pH benchtop meter was used for pH readings. New Brunswick Innova 4,430 shaker incubators were used for bacterial cultivation. Analytical balances from Denver Instruments, a Fisherbrand analog vortex mixer, a Labconco Dry System/Freezone 2.5 lyophilizer, and a Buchi R-200 rotary evaporators were also used. Analytical grade solvents (Fischer and VMR) were used for the isolation and purification procedures. LC-MS grade solvents (Honeywell CHROMOSOLV LC-MS water and Supelco LiChrosolv LC-MS acetonitrile) buffered with formic acid (Millipore Sigma) were used for LC-MS runs. Media were prepared using Millipore Sigma products, except for the pyruvic acid, which was obtained from BeanTown Chemical in MilliQ purified water. Frozen stocks of the strain were stored in a VWR brand ultralow temperature freezer set to −70 °C. For Marfey’s analysis, *N*-(5-flouro-2,4-dinitrophenyl)-L-alaninamide (L-FDAA) was purchased from TCI America, while the following high quality amino/hydroxy acid standards were used: L-(−)-*threo*-3-hydroxyaspartic acid (TOCRIS Bioscience), L-2,4-diaminobutyric acid dihydrochloride (Chemodex), DL-2,4-diaminobutyric acid dihydrochloride (Alfa Aesar), L-ornithine hydrochloride (Acros), D-ornithine hydrochloride (Alfa Aesar), cis-epoxysuccinic acid (TCI America), and (+/−)-trans-oxirane-2,3-dicarboxylic acid (Sigma-Aldrich). The isotope-labeled compounds glycine (^13^C2, 97%–99%) and L-aspartic acid (^15^N, 98%) were purchased from Cambridge Isotope Laboratories Inc.

### 2.2 Media preparation

A modified version of Acidovorax Complex Medium (ATCC Medium 2,688) (Pinel et al., 2008), which does not include yeast extract, was used for eliciting siderophore production, termed “Defined Medium for Siderophores” (DMS). Briefly, the medium was prepared with 0.30 g/L KH_2_PO_4_, 0.30 g/L MgSO_4_, 1.60 mL/L pyruvic acid, 2.00 g/L L-glutamine, and 2.00 g/L 3-(*N*-morpholino)propanesulfonic acid (MOPS) in ∼80% total volume of deionized water that, after being fully dissolved, was brought to pH 7.5 using 1.0 M sodium hydroxide, diluted to the final volume with MilliQ water, and sterilized by autoclaving using a 15 min liquids cycle.

### 2.3 Bacterial sample and cultivation

*Cupriavidus necator* B-4383 was provided by the Agricultural Research Services Culture Collection (ARS) as a lyophilized stock, and, after rehydration and plating on NRRL-1 medium (Agricultural Research Service, n.d.), a single colony was placed into DMS and grown to turbidity. Then, a 1:1 50% glycerol stock was prepared and frozen at −70 °C for long-term storage of the strain. For bacterial cultivation, *C. necator* B-4383 was first restruck from the frozen stock on solid agar plates of DMS (with 15 g/L agar). After sufficient growth, a single colony was then inoculated into a liquid culture at a 5 mL scale; this starter culture was used to inoculate either the biological replicates for the iron supplementation experiment or larger scale media preparations for isolation work.

### 2.4 Iron supplementation experiment

In biological duplicates, 5.0 µL of starter culture was inoculated into 5.0 mL of fresh DMS supplemented with 50 µL of either a filter sterilized solution of 0.12 g citric acid monohydrate in 200 mL deionized water (for the iron-depleted condition) or a filter sterilized solution of 0.12 g citric acid monohydrate and 0.12 g ferric ammonium citrate in 200 mL deionized water (for the iron replete condition). The addition of ferric ammonium citrate solution to the culture broth afforded a final iron concentration of 23 nM. These cultures were grown for 3 days and then centrifuged (6 min, 21,000 rcf at 13 °C) to remove bacterial cells. The supernatants were passed through 100 mg C_18_ RP-SPE cartridges with three elutions of 1,000 µL (MilliQ H_2_O, 50% ACN/H_2_O, and ACN), isolating the siderophores in the 50% ACN/H_2_O fraction. These fractions were run on the LCMS to study metabolomic differences under these two conditions.

### 2.5 Liquid chromatography and mass spectrometry method

LC-HRMS analysis of fractions from the iron supplementation experiment was carried out using an Agilent 6530C Q-TOF LC/MS system with an Agilent jet stream source and an Agilent 1,260 Infinity II pump stack. Chromatography was accomplished using a Core-Shell Kinetex, 2.6 μm, 50 × 2.1 mm, 100 Å EVO C_18_ column (Phenomenex). The LC gradient pump method used 0.1% formic acid (puriss, Sigma Aldrich) and acidified H_2_O (redistilled) as solvent A and 0.1% formic acid (puriss, Sigma Aldrich) and acidified ACN (various suppliers, always LCMS grade) as solvent B, with the following program utilized: a starting elution of 90% solvent A and 10% of solvent B for 3 min, then a linear gradient to 25% solvent B over 5 min, then a linear gradient to 99% solvent B over 7.5 min, then a hold for 3 min at 99% solvent B, and finally, a return to the starting elution over 2 min and a re-equilibration for 2.5 min at a flow rate of 450 μL/min. A 10 μL injection volume was used with the autosampler. The MS method had three time segments; from the start of the procedure to 3.0 min, the flow was diverted to waste to prevent salts from being sprayed on the mass spectrometer, then, from 3.0 to 17.5 min, the flow was sent to the mass spectrometer. At the 17.5-min mark to the end of the run, it was sent back to waste. Centroid data was collected using an Auto MS/MS method, collected in static positive ion polarity with absolute storage thresholds of 200 and 5 for the MS and MS/MS scans, respectively. The source settings were as follows: drying and sheath gas temperatures of 300 °C and 325 °C, respectively; drying and sheath gas flow rates of 10 and 3 L/min, respectively; a nebulizer pressure of 50 psi; and capillary and nozzle voltages of 4,000 and 0 V, respectively. The ion optics were set with a fragmentor at 200 V, the skimmer at 65 V, and the octopole 1 RF Vpp at 750 V. In the experiment segment (from 3.0 to 17.5 min), the Auto MS/MS settings were as follows: an MS scan over the mass range of 100–1,600 *m/z*, with an acquisition rate of 5 spectra/s and an MS/MS scan over the mass range of 100–1,305 *m/z*, with an acquisition rate of 3 spectra/s. The MS/MS collision energy was set at a gradient using the following formula: 3 (*m/z*)/100 + 15*.* For the MS/MS acquisition, a maximum of six precursors per cycle was used, with static exclusion below 250 and above 1,300 *m/z*, or below an absolute threshold of 10,000 counts and active exclusion after 3 spectra within a 0.2-min range. Reference mass signals and some common contaminants were also added to an exclusion list for the entirety of the run (922.0098, 531.40777, 553.38972, and 1,083.791 *m/z* at a range of 100 ppm).

### 2.6 MS/MS data analysis

**FIGURE 1:**
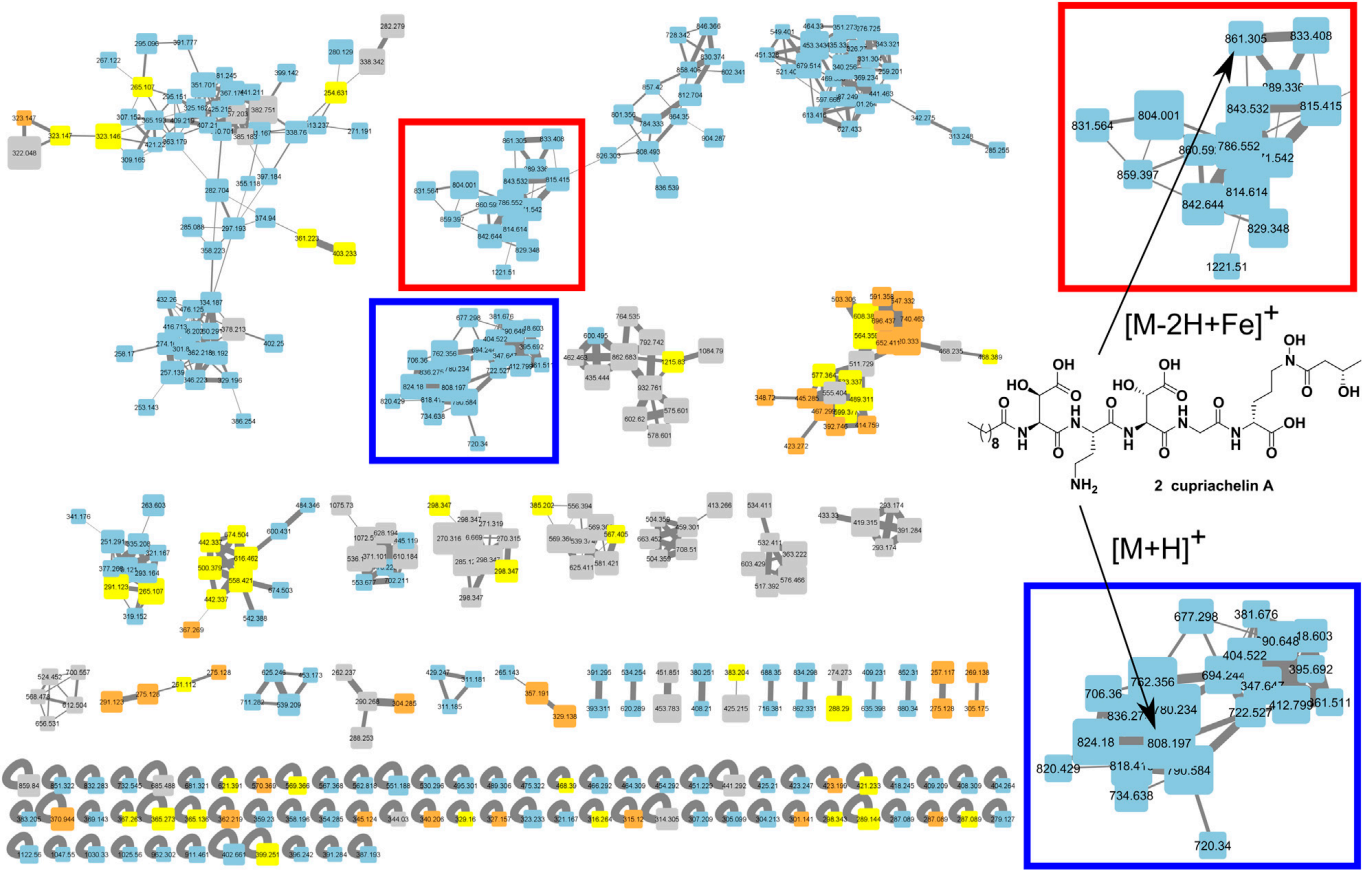
Metabolomic analysis of *C. necator* culture supernatant under low and high iron conditions using the molecular networking tool. This analysis revealed a cluster of putative cupriachelins associated with the [M + H]^+^ ion of the known compound cupriachelin A (blue highlighted section [M + H]^+^ at 808 *m/z*), as well as adducts with the iron cation (red highlighted section [M-2H + Fe]^+^ at 861 *m/z*). Size of nodes based on number of spectra (increasing size with more spectra), color by file source: gray = no injection blank (both from the blank run and masses shared between the blank run and any other run), blue = supernatants from low iron runs, orange = supernatants from high iron runs, and yellow = shared masses between the low and high iron runs (but not the blank).

A molecular network (Figure 1) was created with the duplicate runs from the high/low iron experiment, a run of the strain under low iron from the isolation efforts, and no injection blank runs before each of these experiments using the Global Natural Product Social Molecular Networking web platform (Wang et al., 2016), with a minimum matched fragment ions setting of 6, a minimum cluster size setting of 2, and a cosine score setting of 0.55. We reported the use of MassQL to identify tail length analogs based on predicted changes to fragment ions containing the lipid chain upon adding or removing two carbons to the length of the lipid (Jarmusch et al., 2022); briefly, we used a search where a cupriachelin canonical 523.2358 *m/z* fragment ion (see Supplementary Figure S8) and a fragment bearing the glycine and lipid tail, 212.1645 ± 28.0313 *m/z* (see Supplementary Figure S16), were both present, given a tolerance of 10 ppm and intensity percentage of 5, to identify the presence of **4-6** in our datasets. Manual annotation of all fragment spectra was carried out to validate these assignments, shown in Supplementary Figure S7–Supplementary Figure S40 and Supplementary Table S3–Supplementary Table S19.

### 2.7 Siderophore isolation

For siderophore isolation, 500 µL of the starter culture was added to 2 × 1 L flasks (each containing 500 mL of liquid DMS). The bacterium was allowed to grow shaking at 180 RPM on a rotary shaker at 30 °C for approximately 48 h. The culture flasks were harvested by shaking them with HP-20 resin (20 g/L at 180 RPM for 2 h using an orbital shaker). This suspension was filtered through filter paper to remove the culture supernatant and cells; then, the remaining resin was washed with 0.5 L of water. Finally, the adsorbed metabolites were eluted using 4 × 100 mL of methanol. This methanol extract was concentrated by rotary evaporation, and the presence of the siderophores was confirmed using LC-MS analysis. The crude extract (132 mg) was subsequently cleaned by first passing it through a Sephadex LH-20 column with an isocratic methanol elution, collecting fractions of ca. 50 mL to remove media components. LC-MS analysis revealed fractions containing the siderophores; these fractions were again dried by rotary evaporation and further purified by RP-SPE with a 5 g C_18_ column using 20 mL elutions of, sequentially, H_2_O, 50% ACN/H_2_O, and then ACN. The 50% ACN/H_2_O fraction was purified via RP-HPLC using a C_18_ semipreparative (Phenomenex Luna^®^, 250 × 10 mm, 5 μm) column and a 2.75 mL/min flow rate with a linear gradient from 50% ACN/H_2_O acidified with 0.1% formic acid to 90% ACN/H_2_O acidified with 0.1% formic acid over 35 min to allow isolation of pure compounds. Fractions were collected in even 1.5 mL volumes and peaks identified by the 210 nm chromatogram were checked for purity using LC-MS analysis before being combined, concentrated to reduced volume by rotary evaporation, and then reduced to dryness in a freeze dryer.

*Cupriachelin B* (**1**): Yellowish white powder [α]^22.6^_D_ −22.5 (*c* = 0.4, H_2_O); UV (H_2_O) λ_max_ was obscured by solvent absorption (<200 nm) and could not be reliably measured; IR_*νmax*_ 3,300, 2,924, 1,638, 1,436, 1,107cm^−1^; ^1^H NMR (500 MHz, D_2_O), ^13^C NMR (125 MHz, D_2_O), and 2D NMR data are shown in Supplementary Table S2; HRESIMS (positive mode) *m/z* 780.3619 [M + H]^+^ (calcd. for C_31_H_54_N_7_O_16_^+^ [M + H]^+^, 780.3622, 0.4 ppm).

### 2.8 Amino acid configuration by Marfey’s analysis

For siderophore hydrolysis, circa 2 mg of **1** was dissolved in 200 μL of MilliQ water, followed by the addition of 200 μL of 55% HI. The acidified solution was then transferred to a one-dram vial and the cap was sealed tightly with parafilm. The sealed vessel was heated for 22 h at 100 °C; then, the crude hydrolysate was transferred to a fresh vial, which was evaporated using a nitrogen gas flow. The dried material was repeatedly redissolved in ∼700 μL of MilliQ water (three times total) to remove any residual acid and then brought to a final volume of 100 μL in MilliQ water. The hydrolysate was reacted with L-FDAA (Marfey’s reagent) under conditions mentioned in the literature (Reitz et al., 2019).

### 2.9 *β*-Hydroxyaspartic acid diastereomer synthesis

Diastereomeric mixtures of DL-*threo*-*β*-hydroxyaspartic acid and DL-*erythro*-*β*-hydroxyaspartic acid were prepared by treatment of *cis*-epoxysuccinic acid (0.2 g) and *trans*-epoxysuccinic acid (0.2 g) with 5 mL of a concentrated (28%) aqueous solution of NH_4_OH (Acros Organics) following a previously described method (Robertson et al., 2018). Reactions were allowed to proceed for 24 h at 50 °C. Then, the reaction mixtures were dried *in vacuo*, yielding thick syrups. The mixtures were dissolved in 10 mL of H_2_O and acidified to pH 3 with concentrated HCl. The solution of D/L-*threo*-*β*-hydroxyaspartic acid was maintained at 4 °C over a 72-h period, after which a white precipitate was collected by filtration and washed with cold H_2_O (100 mL) and dried *in vacuo*. This material was then frozen and residual water was dried by lyophilization, yielding 150 mg as thin colorless needles. This material was used without further purification. The D/L-*erythro*-*β*-hydroxyaspartic acid mixture did not recrystallize well in our hands; therefore, the dried colorless crude reaction product was purified using a 1,000 mg C_18_ RP-SPE cartridge in which the D/L-*erythro*-*β*-hydroxyaspartic acid was eluted with 25% ACN in H_2_O. This fraction was evaporated and used without further purification.

### 2.10 Photoreactivity test of the cupriachelins SPE fraction

A 50% ACN in H_2_O eluent was used to purify the cupriachelins from the *C. necator* supernatant extract on a C_18_ RP-SPE column (see above). In this experiment, this cupriachelins fraction was dried under vacuum; then, in duplicate, a ∼1 mg/mL solution of the SPE fraction in PBS buffer (pH 7.5) was prepared in a clear glass dram vial and exposed to natural sunlight for 6 h. Identical solutions that were shielded from sunlight by wrapping the vials in aluminum foil served as negative controls. After light exposure, samples were centrifuged to remove any trace particulates and the supernatants were run on the LC-MS to identify candidate degradation products, using the same method as described above.

For the isotope-labeled experiments, 5 mL scale cultures were grown in DMS supplemented with either L-aspartic acid and glycine (the negative control), ^15^N-L-aspartic acid and glycine, or L-aspartic acid and (1,2)-^13^C-glycine. Briefly, a stock solution of each amino acid at a concentration of 100 mM in MilliQ purified water was filter sterilized and introduced to the cultures at a dilution of x25, resulting in a final concentration of 4 mM. Bacteria were cultured in these media for 2 days and then centrifuged to collect clear supernatants that were passed through SPE columns and the 50% ACN in H_2_O was collected and dried (as above). This material was resuspended in 1 mL of PBS and 50 μL of ferric ammonium citrate stock (a filter sterilized solution of 0.12 g citric acid monohydrate and 0.12 g ferric ammonium citrate dissolved in 200 mL of MilliQ purified water) was added and the samples were split, with one vial being wrapped in aluminum foil before being left in direct sunlight for 3 h. After incubation all samples were centrifuged to remove any particulates and run on the LCMS, injecting 20 μL using the method discussed above.

### 2.11 Broth dilution antifungal susceptibility testing

To determine the antifungal activity of the *C. necator* extract and compound **1**, the following strains were used: *C. albicans* ATCC 90028, *C. neoformans* ATCC 90113, and *A. fumigatus* ATCC 204305. All these strains are purchased from the American Type Culture Collection (ATCC, Manassas, VA). Susceptibility testing was performed using a modified version of the CLSI methods (Clinical and Laboratory Standards Institute, 2002; Clinical and Laboratory Standards Institute, 2008). Briefly, all samples were serially diluted in 20% DMSO/saline and transferred in duplicate to 384 well flat-bottom microplates, maintaining a final DMSO concentration of 1% in the assay. Inocula were prepared by correcting the OD_630_ of microbe suspensions in the incubation broth by following the McFarland standard. The incubation media were RPMI 1640 (2% dextrose/0.03% glutamine/MOPS at pH 6.0) for *C. albicans*, Sabouraud Dextrose for *C. neoformans*, and RPMI 1640 broth (2% dextrose, 0.03% glutamine, buffered with 0.165 M MOPS at pH 7.0) for *A. fumigatus*. 5% Alamar Blue™ was added to *A. fumigatus*. Drug controls for fungi were included in this assay. The optical density was read using a Bio-Tek plate reader prior to and after incubation: *C. albicans* and *A. fumigatus* at 35 °C for 48 h and *C. neoformans* at 35 °C for 68–72 h. The concentration of compound **1**/crude extract responsible for 50% growth inhibition (IC_50_) was calculated using XLfit 4.2 software (IDBS, Alameda, CA), employing the fit model 201.

## 3 Results and discussion

### 3.1 Metabolomic comparison of *C. necator* B-4383 under iron deficient and enriched conditions

The bacterium was grown on a defined medium termed “Defined Medium for Siderophores” (DMS), which was modified from Acidovorax Complex Medium (ATCC Medium 2,688) (Pinel et al., 2008). Cultures were supplemented with either ferric ammonium citrate in a citric acid solution or a solution of citric acid alone. After 3 days of culturing, supernatants were fractioned using reverse phase solid phase extraction (RP-SPE) and then both processed supernatants underwent LCMS profiling to reveal siderophore production. Unsurprisingly, the LC-MS results showed characteristic masses (e.g., the known cupriachelin A [M + H]^+^ ion at 808 *m/z*) in the iron-limited condition, while these masses were lacking with iron supplementation. These data were further analyzed using the Global Natural Product Social Molecular Networking platform (GNPS) (Wang et al., 2016) and visualized with Cytoscape to see if these masses clustered with, and could be expected to share structural similarity with, the known cupriachelins. Molecular networking helped us to characterize a cupriachelin cluster with many masses unique to the iron-limited condition clustering with the presumed cupriachelin A mass at 808 *m/z* (Figure 1). The results warranted further investigation of the MS/MS and fragmentation patterns to assign putative structures to this cluster.

### 3.2 Annotation of MS/MS fragmentation spectra to identify cupriachelin congeners

In annotating the presumed cupriachelin cluster detected in the supernatant of the *C. necator* B-4383 culture under iron limitation, masses that possessed distinct retention times, and were likely not ionization-derived source fragments of more abundant compounds, were noted, resulting in a table of 17 candidate compounds (SM Table 1). This search was followed by analysis of the MS/MS fragmentation pattern of **2**, the presumed cupriachelin A [M + H]^+^ precursor mass (808 *m/z*), which showed agreement with cupriachelin A fragments (Kreutzer et al., 2012) (Supplementary Figure S9 and Supplementary Figure S10 and Supplementary Table S4), helping to confirm our hypothesis that this strain produces cupriachelins.

**FIGURE 2:**
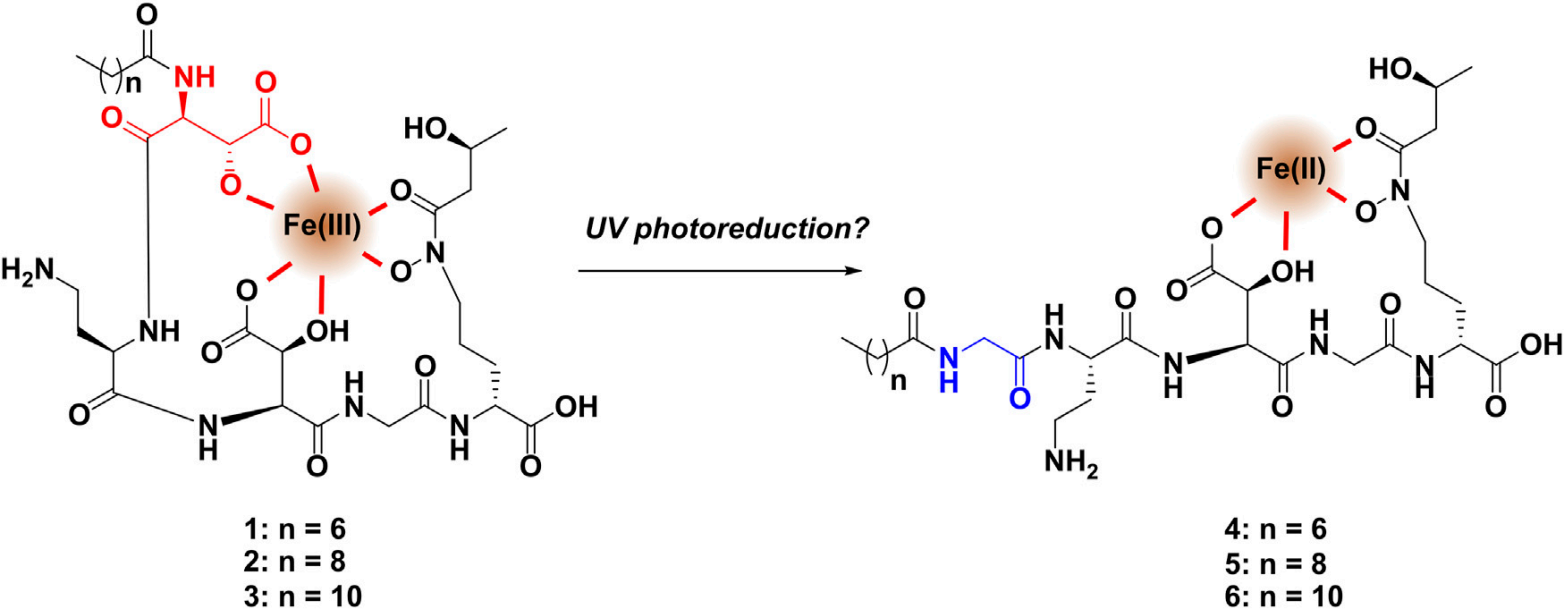
With only one *β*-hydroxyaspartic acid, the binding to ferric iron of compounds **4-6** is presumed to be far weaker than that of the major compounds **1-3**, which may explain why iron adducts of **4-6** were not observed in the molecular network.

Using the annotated MS/MS fragmentation spectrum of cupriachelin A as a template, the fragment spectra of other candidate cupriachelin analogs, compounds **1** and **3–17**, were annotated. The modifications include changes to the lipid tail, as was observed prior in *C. necator* H16 (except for compound **3**) (Kreutzer et al., 2012). An abundant analog that drew our attention was a compound with an [M + H]^+^ at 780 *m/z*, as this was 28 Da less than the precursor mass of **2**, and, knowing that changes in lipid tail are common, it was hypothesized that this was an analog with an eight-carbon lipid tail. Annotation of the MS/MS spectrum supported this as the correct assignment for compound **1** (Supplementary Figure S7 and Supplementary Figure S8 and Supplementary Table S3). We also annotated the analog with a twelve-carbon lipid tail (compound **3**, Supplementary Figures S11, S12 and Supplementary Table S5), but it was less abundant in the extract. Several dehydration events (compounds **7–10**), which may be extraction artifacts, were detected, often only as trace constituents of the concentrated fractions during HPLC purification (Supplementary Figure S19–Supplementary Figure S26 and Supplementary Tables S9–Supplementary Table S12). A methyl ester (compound **17**), which is almost certainly an extraction artifact from methanol, was also detected during some large-scale preaparatirfvons (Supplementary Figure S39 and Supplementary Figure S40 and Supplementary Figure S45). With the formation of methyl esters from carboxylic acids in methanol being an equilibrium process, any time methanol is used during the extraction process (as we did here), formation of methyl esters from carboxylic acids should be expected to at least some small extent (Maltese et al., 2009; Venditti, 2020); our confidence in assigning **17** as an artifact comes from the predominance of the carboxylate product and prior genomic investigations that did not reveal methyl transferases installing methyl esters in related siderophores (Reitz et al., 2019). Compounds **11** and **12** show the incorporation of aspartic acid (a proteogenic amino acid) rather than *β*-hydroxyaspartic acid. We hypothesize that they are likely to arise from enzyme promiscuity in the biosynthetic assembly line, incorporating aspartic acid rather than *β*-hydroxyaspartic acid, leading to these minor analogs at low titer. The abiotic loss of water from a *β*-hydroxyaspartic acid via dehydration in **1-3** after biosynthesis would instead give a compound similar to **10**, and cannot explain **11** and **12**. In compounds **4-6,** the first *β*-hydroxy aspartic acid in the peptide backbone was instead observed to be glycine (Supplementary Figure S13–Supplementary Figure S18 and Supplementary Tables S6–8). These compounds could be identified with the help of shared fragment ions containing the glycine residue and the lipid tail, as we reported using the MassQL search tool (SM Figures 14, 16, and 18) (Jarmusch et al., 2022). Lacking the first *β*-hydroxy-aspartic acid, these analogs are presumed to have a lower metal binding activity (Figure 2), as this residue is a key feature in the iron binding of many known siderophores (Reitz et al., 2019). In our network, masses adducted to iron were detected, with ferric iron bound to compounds **1–3** (Supplementary Figure S46 and Supplementary Figure S47 and Supplementary Table S21), and no masses consistent with the iron adducts for compounds **4-6** were detected, supporting this hypothesis. Precursor masses consistent with the iron adducts of **7-9** were detected but these may be source fragments from ionization of the iron adducts of **1-3**, and therefore, the spectra were not annotated.

The production of compounds **4-6** was at first thought to be temperamental, with these metabolites not always found in LC-MS analysis of the fermentation broth. However, in a UV exposure experiment in the presence of ferric iron, these masses were detected at very low titer along with a suite of novel smaller degradation products only in the UV-exposed samples but not in the control that was protected from UV exposure. Among the minor metabolites, we annotated three compounds (**18–20**) that bear the glycine residue of **4-6** but appear to have fragmented after the L-diaminobutyrate (L-DAB) residue (Supplementary Figure S42 and Supplementary Figure S43 and Supplementary Table S20). Given the results of this experiment, we suspect that these molecules are byproducts of a reductive reaction on ferric iron by the major cupriachelins (Figure 2). The reason why these analogs were not consistently formed in the fermentation broth, even though DMS does not include an iron source, is that in some batches of the media, small amounts of contamination by ferric iron could lead to this conversion via a reductive reaction with the metal ion. The UV exposure experiment was conducted to replicate the work of Kreutzer and coworkers, who noted degradation of the cupriachelins to a truncated aldehyde product via the photoreduction of ferric iron in sunlight, which the authors hypothesized could have an ecological significance in terms of iron acquisition (Kreutzer et al., 2012). However, as we were not able to detect their reported aldehyde-containing cupriachelin degradation product, the aldehyde may have been unstable and may have further degraded during the course of the experiment. Repeating the UV exposure experiment with material collected from a culture grown on (1,2)^13^C-labeled glycine or ^15^N-labeled aspartic acid showed masses consistent with the glycine analogs (**4–6**) coming from the degradation of hydroxyaspartic acid into glycine, i.e., the reduction shown in Figure 2 (Supplementary Figure S44 and Supplementary Figure S45).

The dominant lipid tail length appears to distinguish the metabolomes of *C. necator* H16 and B-4383; in *C. necator* B-4383, the most abundant species is the cupriachelin analog with an eight-carbon lipid tail (**1**), while in *C. necator* H16, the ten-carbon analog (**2**) was reported to be dominant (Kreutzer et al., 2012).

### 3.3 Scaling-up the production for purification and isolation of major cupriachelin analogs

The crude extract led to two major compounds, which we were able to isolate at milligram scale. The first major metabolite (**1**), with an [M + H]^+^ precursor ion at 780 *m/z*, was analyzed using 1D- and 2D-NMR and then compared with the previously reported data of cupriachelin A (**2**) (Kreutzer et al., 2012), and we arrived at our candidate structure from annotation of the HRMS fragment spectrum. The 2D-NMR data of **1** matched well with the reported data of cupriachelin A (**2**). The HSQC spectrum of **1** (SM Figure 4 and Table 2) was inspected for diagnostic correlations between the protons and α-carbons of the first hydroxyaspartic acid (δ_H_ 4.61, δ_C_ 55.6), the DAB (δ_H_ 4.54, δ_C_ 50.7), the second hydroxyaspartic acid (δ_H_ 4.98, δ_C_ 56.0), the downfield methylene of glycine (δ_H_ 3.98, δ_C_ 41.6), and *N* ^δ^-acyl-*N* ^δ^-hydroxyornithine (δ_H_ 4.27, δ_C_ 52.8). Also expected in this region were cross peaks for the oxymethines of the *β*-hydroxyaspartic acids, which were observed at δ_H_ 4.33, δ_C_ 70.8 (for the first residue) and δ_H_ 4.58, δ_C_ 70.5 (for the second residue), as well as the oxymethine of the hydroxybutyric acid (seen at δ_H_ 4.22, δ_C_ 63.6) and the methylene in the δ-position in *N* ^δ^-acyl-*N* ^δ^-hydroxyornithine (seen at δ_H_ 3.70–3.62, δ_C_ 46.3), which was shifted downfield by the hydroxyacylamine. All these shifts matched well to the known spectra of **2** (Supplementary Table S22). However, the fragmentation pattern suggested a loss of two carbons from the lipid tail. Close inspection showed that, within the overlapping HSQC signals for the internal carbons on the lipid tail, this analog has two fewer carbon species in the 1D-NMR (Supplementary Figure S3 and Supplementary Figure S4 and Supplementary Table S2). Cupriachelin A (**2**) was isolated as the second most abundant compound.

### 3.4 Stereochemical assignment

Amino acid configurations of **1** were determined by Marfey’s analysis (Marfey, 1984; Bhushan and Brückner, 2004). Compound **1** was hydrolyzed with 55% HI and then treated with L-FDAA, as described previously (Reitz et al., 2019). The hydrolysate was compared to L-FDAA-derivatized amino acid standards of known configurations. Analysis established the configurations of the residues within **1** as L-*threo*-*β*-hydroxyaspartic acid, L-*erythro*-*β*-hydroxyaspartic acid, L-DAB, and D-ornithine. Standards of D/L-*threo*-*β*-hydroxyaspartic acid and D/L-*erythro*-*β*-hydroxyaspartic acid were prepared from *cis*-epoxysuccinic acid and *trans*-epoxysuccinic acid, respectively, according to a literature protocol (Jones et al., 1969). D/L-*threo*-, D/L-*erythro*-*β*-hydroxyaspartic acids, and a pure compound standard of L-*threo*-*β*-hydroxyaspartic acid were then derivatized with L-FDAA and compared to the LC-HRMS profile of the L-FDAA-treated HI hydrolysate. The hydrolysate contained two peaks with the expected mass, and the first hydrolysate peak matched the retention time of the L-*threo*-*β*-hydroxyaspartic acid standard (4.62 min). The second peak in the hydrolysate had the same retention time (9.14 min) as the later elution of the two *erythro*-*β*-hydroxyaspartic acid diastereomer peaks (Supplementary Figure S41). It was previously established that the elution order with C_18_ HPLC conditions for L-FDAA-derivatized *erythro*-*β*-hydroxyaspartic acid diastereomers was D followed by L (Fujii et al., 1997). The assignment of *β*-hydroxyaspartic acids as L-*threo* and L-*erythro* was consistent with the revision to the structure of cupriachelin A by Reitz and coworkers (Reitz et al., 2019). Importantly, our assignment of D-ornithine does not match the original report by Kreutzer and coworkers (Kreutzer et al., 2012) or the structure shown by Reitz and coworkers, although the latter reassignment did not include any analysis of the ornithine residue, only the *β*-hydroxyaspartic acids (Reitz et al., 2019). The stereochemistry of 3-hydroxybutyric acid represented in our figures is based on the original assignment of cupriachelin A (Kreutzer et al., 2012).

### 3.5 Antifungal bioassay

Both a resin extract of *C. necator* B-4383 culture broth and compound **1** showed activity against *C. neoformans*, exhibiting IC_50_ values of 16.6 and 3.2 μg/mL, respectively, while no activity was detected toward *C. albicans* or *A. fumigatus*. This activity is likely due to the critical role of the iron acquisition pathway in the growth and pathogenesis of *C. neoformans* (Won et al., 2006; Jung et al., 2009; Saikia et al., 2014). Nevertheless, iron acquisition also plays a crucial role in the growth and survival of other organisms such as *A. fumigatus* and *C. albicans* (Almeida et al., 2009; Brandon et al., 2015). This lack of antifungal activity in both *A. fumigatus* and *C. albicans* may be attributed to differences in the specificity of the siderophore membrane transporter systems belonging to each of these organisms, which may not allow *C. neoformans* to appropriate the cupriachelin siderophore (Crowley et al., 1991; Haas et al., 2003). Previous studies have demonstrated the antifungal potential of other siderophores (e.g., enterobactin) against the *Aspergillus* and *Candida* genera (Sheng et al., 2020; Khan et al., 2021), reinforcing the hypothesis that the observed difference in antifungal activity is not related to iron sensitivity *per se*, but the ability to transport the exogenous cupriachelin siderophores. As a control, the lyophilized residue of sterile DMS and ethylenediaminetetraacetic acid (EDTA) were submitted to the same panel, and the media showed no activity at the highest concentration evaluated (200 μg/mL), while EDTA had an IC_50_ for *C. albicans* of 7.3 μg/mL and for *C. neoformans* of 13.1 μg/mL (Table 1). Compound **1** was not active against methicillin-resistant *Staphylococcus aureus*, *Escherichia coli*, *Pseudomonas aeruginosa*, *Klebsiella pneumoniae*, or vancomycin-resistant *Enterococcus faecium* at the highest dose evaluated (200 μg/mL, data not shown).

**TABLE 1:** Antifungal activity results.

Sample	IC_50_ (μg/mL)		
	*C. neoformans* ATCC 90113	*A. fumigatus* ATCC 204305	*C. albicans* ATCC 90028
*C. necator* resin extract	16.6	>200	>200
Compound **1**	3.2	>20	>20
EDTA	13.1	>20	7.3
Lyophilized DMS	>200	>200	>200

Note: where the IC_50_ values were above the maximum concentration evaluated, the values are reported only as a range (e.g., >200 μg/mL).

## 4 Conclusion

Our investigation into *C. necator* B-4383 revealed the production of cupriachelin siderophores, building on prior research that showed that this species produces reactive siderophores that aid in surviving iron limitation (Kreutzer et al., 2012). In our metabolomic investigation, we show differences between the preferred lipids incorporated into the cupriachelins by strain H16 *versus* B-4383 and identify, at low titer, evidence for an interesting degradation of hydroxy aspartic acid into glycine; something that we will investigate in other siderophores. We also report the activity of compound **1** against the fungal pathogen *C. neoformans* for the first time.

## Data Availability

The LCMS data for the iron limitation experiment, the UV exposure experiments, and the crude extract of the large scale for compound isolation have been uploaded to the GNPS-MassIVE archive with accession ID: MSV000092364. Processed NMR data has been uploaded to the eGROVE archive as a Mnova file with the following permanent link: https://egrove.olemiss.edu/pharmacy_facpubs/132.
