# Supplementary material for: Comparative metabolomic profiling of Cupriavidus necator B-4383 revealed production of cupriachelin siderophores, one with activity against Cryptococcus neoformans
Source: Front Chem. 2023 Aug 24;11:1256962. doi: 10.3389/fchem.2023.1256962 (PMC10484230; doi:10.3389/fchem.2023.1256962)
Supplement: Supplementary file 1 [file DataSheet1.docx]

Supplementary Material

Comparative Metabolomic Profiling of *Cupriavidus necator* B-4383 Revealed Production of Cupriachelin Siderophores, One with Activity Against *Cryptococcus neoformans*

Mohammed M. A. Ahmed^1,2^, Siddarth K. Tripathi^3^, Paul D. Boudreau^1*^

^1^Boudreau Lab, Department of BioMolecular Sciences, School of Pharmacy, University of Mississippi, University, MS, USA

^2^Department of Pharmacognosy, Al-Azhar University, Cairo, Egypt

^3^National Center for Natural Products Research, School of Pharmacy, University of Mississippi, University, MS, USA

*** Correspondence:**Corresponding Author
[boudreau@olemiss.edu](mailto:boudreau@olemiss.edu)

Table of Contents

1 Supplementary Figures and Tables

## 1.1. IR and NMR spectra and summary table for compound 1………………………….2

## 1.2. HRMS spectra, fragment structures, and summary tables of compounds 1-17…….7

## 1.3. Marfey’s analysis of compound 1…………………………………………………..28

## 1.4. HRMS fragment spectra, fragment annotations, and summary tables from the UV exposure experiments………………………………………………………………………...29

## 1.5. Annotation of iron adducts of compounds 1-3…………………………..…………33

## 1.6. NMR shift comparison of cupriachelin A and compound 1………………………..36

# Supplementary Figures and Tables

## IR and NMR spectra and summary table for compound 1.


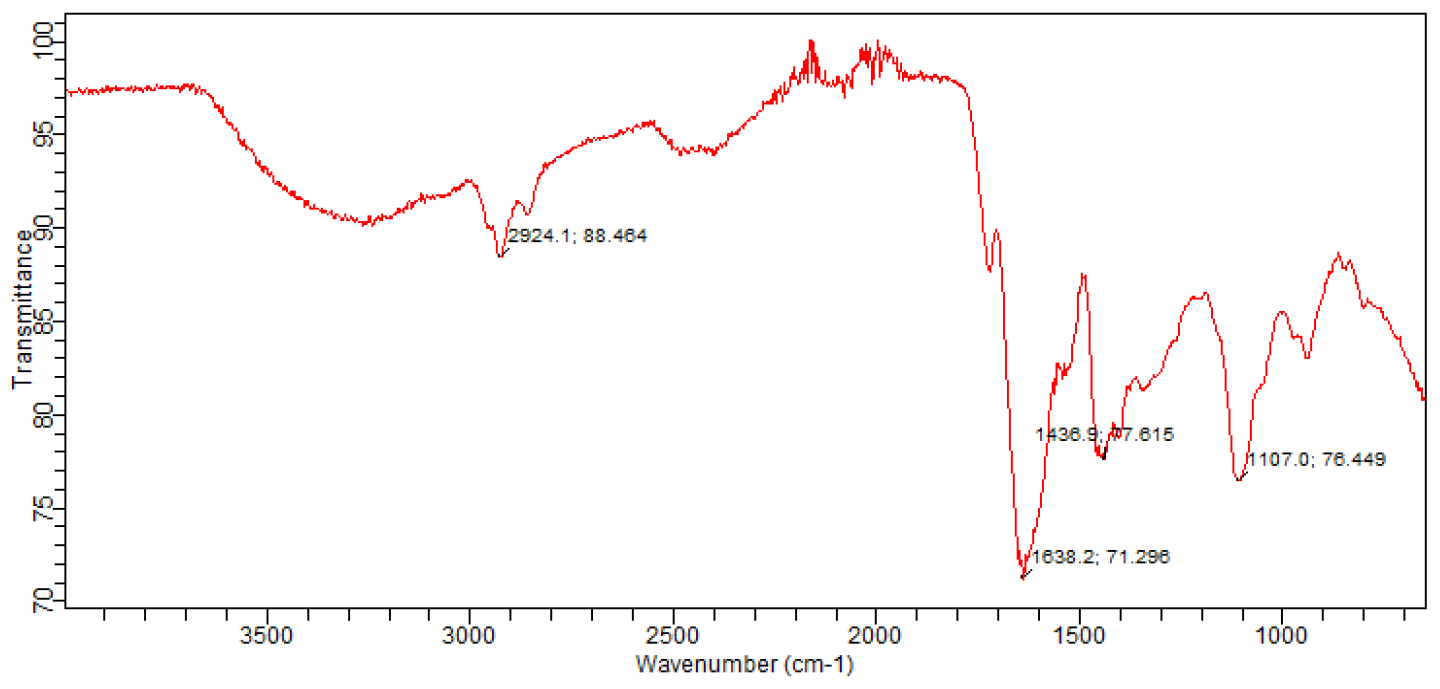
**Supplementary Figure 1.** The IR spectrum of compound **1**.

**Supplementary Figure 2.** ^1^H-NMR (500 MHz, D_2_O) spectrum of compound **1**, referenced to TMS at δ_H_ 0.0.

**Supplementary Figure 3:** ^13^C-NMR (125 MHz, D_2_O) spectrum of compound **1**.

**Supplementary Figure 4:** HSQC-NMR (500 MHz, D_2_O) spectrum of compound **1**.

**Supplementary Figure 5:** HMBC-NMR (500 MHz, D_2_O) spectrum of compound **1**.

**Supplementary Figure 6:** HMBC correlations observed in compound **1**.

| Residue | Position | δ_C_ | δ_H_, M (*J* in Hz) | HMBC |
| --- | --- | --- | --- | --- |
| Octanoic acid | C-1 | 176.6, C | -- |  |
|  | C-2 | 34.3, CH_2_ | 2.34, t (7.4) | (Octanoic acid) C-1, C-3, C-4 |
|  | C-3 | 24.1, CH_2_ | 1.61-1.578, m | (Octanoic acid) C-4 |
|  | C-4 | 27^a^, CH_2_ | 1.29, m^e^ |  |
|  | C-5 | 27^a^, CH_2_ | 1.29, m^e^ |  |
|  | C-6 | 29.8, CH_2_ | 1.26, m^e^ |  |
|  | C-7 | 20.9(0), CH_2_ | 1.27, m^e^ |  |
|  | C-8 | 12.3, CH_3_ | 0.85, t (6.6) | (Octanoic acid) C-6, C-7 |
| L-*erythro*-*β*-OH-Asp-1 | NH | -- | ND** |  |
|  | C-1 | 171.6, C | -- |  |
|  | C-2 | 55.6, CH | 4.61, m^f^ | (L-*threo*-*β*-OH-Asp-1) C-1 |
|  | C-3 | 70.8, CH | 4.33, m^g^ |  |
|  | C-4 | * | -- |  |
| L-Dab | NH(1) | -- | ND** |  |
|  | C-1 | * | -- |  |
|  | C-2 | 50.7, CH | 4.54, m^f^ | (L-Dab) C-3 |
|  | C-3_a_ | 27.1(5)^b^, CH_2_ | 2.30-2.17, m |  |
|  | C-3_b_ | 27.1(5)^b^, CH_2_ | 2.11-2.06, m |  |
|  | C-4 | 35.2, CH_2_ | 3.11-3.06, m |  |
|  | NH(2) | -- | ND** |  |
| L-*threo*-*β*-OH-Asp-2 | NH | -- | ND** |  |
|  | C-1 | * | -- |  |
|  | C-2 | 56.0, CH | 4.86 broad singlet |  |
|  | C-3 | 70.5, CH | 4.58, m^f^ |  |
|  | C-4 | * | -- |  |
| Gly | NH | -- | ND** |  |
|  | C-1 | 169.7, C | -- |  |
|  | C-2 | 41.6, CH_2_ | 3.98, s | (Gly) C-1 |
| D-N^δ^-OH-Orn | NH | -- | ND** |  |
|  | C-1 | * | -- |  |
|  | C-2 | 52.8, CH | 4.27, m^g^ |  |
|  | C-3_a_ | 27.4^c^, CH_2_ | 1.87-1.83, m |  |
|  | C-3_b_ | 27.4^c^, CH_2_ | 1.71, m^h^ |  |
|  | C-4 | 21.4, CH_2_ | 1.67, m^h^ |  |
|  | C-5 | 46.3, CH_2_ | 3.70-3.62, m |  |
| L-Hbu | C-1 | 172.2, C | -- |  |
|  | C-2_a_ | 39.7^d^, CH_2_ | 2.78, dd (7.8, 14.8) | (L-Hbu) C-1, C-3, C-4 |
|  | C-2_b_ | 39.7^d^, CH_2_ | 2.61, dd (5.5, 14.7) | (L-Hbu) C-1, C-3 |
|  | C-3 | 63.6, CH | 4.22, m^g^ |  |
|  | C-4 | 20.8(7), CH_3_ | 1.23, d (6.3) | (L-Hbu) C-2, C-3 |

**Supplementary Table 2:** NMR data annotation of compound **1**. (*) The carbon signals at 170.7(6) and 170.7(1) could not be assigned via HMBC correlations, though we noted that our carbon spectrum was missing expected carbonyl signals, likely due to either the limited about of material collected or closely related species that our spectrometer could not resolve. (**) In the proton spectrum, exchange with the deuterated water meant that no NH signals were observed. (^a^) HSQC assignment could not differentiate between the two carbon signals at 27.0 and 27.1(0) for these two peaks. (Continued next page.) (^b,c,d^) Diasterotopic protons were split for this position. (^e^) Signals overlap in the multiplet at 1.26-1.19, assignments made by HSQC. (^f^) Signals overlap in the multiplet at 4.56-4.46, assignments made by HSQC. (^g^) Signals overlap in the multiplet at 4.31-4.13, assignments made by HSQC. (^h^) Signals overlap in the multiplet at 1.69-1.59, assignments made by HSQC.

## HRMS spectra, fragment structures, and summary tables of compounds 1-17.


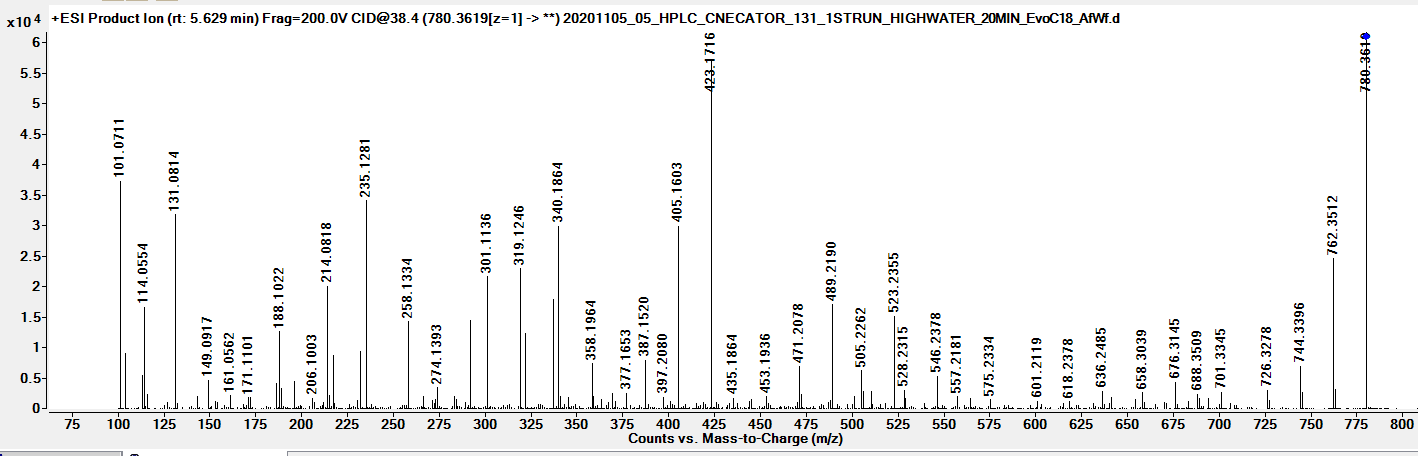


**Supplementary Figure 7:** HRMS^2^ fragment spectrum of compound **1**.

**Supplementary Figure 8:** Annotation of fragments for compound **1**.

| **Fragment calculated formula and *m/z*** | **Observed HRMS/MS *m/z*** | **Error (ppm)** |
| --- | --- | --- |
| C_31_H_52_N_7_O_15_^+^, 762.3516 | 762.3512 | 0.5 |
| C_31_H_50_N_7_O_14_^+^, 744.3410 | 744.3396 | 1.9 |
| C_31_H_48_N_7_O_13_^+^, 726.3305 | 726.3278 | 3.7 |
| C_27_H_46_N_7_O_13_^+^, 676.3148 | 676.3145 | 0.4 |
| C_27_H_44_N_7_O_12_^+^, 658.3042 | 658.3039 | 0.4 |
| C_23_H_38_N_7_O_14_^+^, 636.2471 | 363.2485 | -2.2 |
| C_23_H_36_N_7_O_13_^+^, 618.2366 | 618.2378 | -1.9 |
| C_23_H_33_N_6_O_13_^+^, 601.2100 | 601.2119 | -3.1 |
| C_22_H_36_N_5_O_11_^+^, 546.2406 | 546.2378 | 5.1 |
| C_22_H_34_N_5_O_10_^+^, 528.2300 | 528.2315 | -2.8 |
| C_19_H_35_N_6_O_11_^+^, 523.2358 | 523.2355 | 0.6 |
| C_19_H_33_N_6_O_10_^+^, 505.2253 | 505.2262 | -1.8 |
| C_20_H_33_N_4_O_10_^+^, 489.2191 | 489.2190 | 0.2 |
| C_20_H_31_N_4_O_9_^+^, 471.2086 | 471.2078 | 1.7 |
| C_20_H_29_N_4_O_8_^+^, 453.1980 | 453.1936 | 9.7 |
| C_15_H_27_N_4_O_10_^+^, 423.1722 | 423.1716 | 1.4 |
| C_15_H_25_N_4_O_9_^+^, 405.1616 | 405.1603 | 3.2 |
| C_15_H_23_N_4_O_8_^+^, 387.1510 | 387.1520 | 2.5 |
| C_16_H_28_N_3_O_6_^+^, 358.1973 | 358.1964 | 2.5 |
| C_16_H_26_N_3_O_5_^+^, 340.1867 | 340.1864 | 0.9 |
| C_11_H_19_N_4_O_7_^+^, 319.1248 | 319.1246 | 0.6 |
| C_11_H_17_N_4_O_6_^+^, 301.1143 | 301.1136 | 2.3 |
| C_11_H_22_N_3_O_6_^+^, 292.1503 | 292.1489 | 4.7 |
| C_11_H_20_N_3_O_5_^+^, 274.1397 | 274.1393 | 1.5 |
| C_12_H_20_NO_5_^+^, 258.1336 | 258.1334 | 0.8 |
| C_9_H_19_N_2_O_5_^+^, 235.1288 | 235.1281 | 2.9 |
| C_8_H_14_N_3_O_5_^+^, 232.0928 | 232.0912 | 6.9 |
| C_8_H_12_N_3_O_4_^+^, 214.0822 | 214.0818 | 1.9 |
| C_7_H_14_N_3_O_3_^+^, 188.1030 | 188.1022 | 4.2 |
| C_5_H_11_N_2_O_2_^+^, 131.0815 | 131.0814 | 0.8 |
| C_5_H_8_NO_2_^+^, 114.0550 | 114.0554 | -3.5 |
| C_4_H_9_N_2_O^+^, 101.0709 | 101.0711 | -1.9 |

**Supplementary Table 3:** Summary of selected fragment ions of compound **1**.


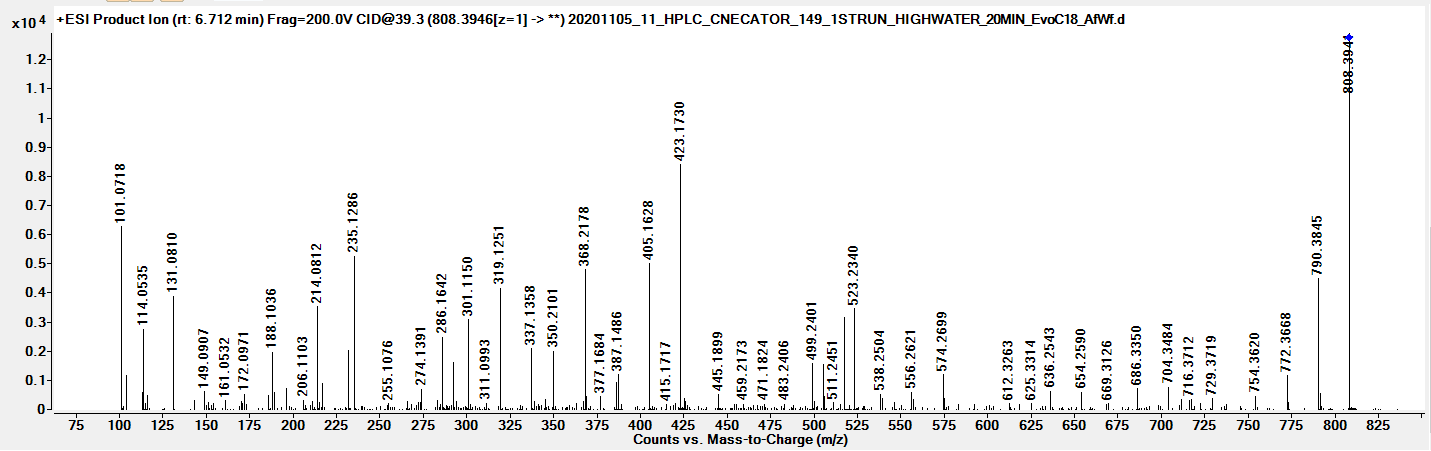


**Supplementary Figure 9:** HRMS^2^ fragment spectrum of compound **2**.

**Supplementary Figure 10:** Annotation of select fragment ions of compound **2**.

| **Fragment calculated formula and *m/z*** | **Observed HRMS/MS *m/z*** | **Error (ppm)** |
| --- | --- | --- |
| C_29_H_50_N_7_O_13_^+^, 704.3461 | 704.3484 | -3.2 |
| C_29_H_48_N_7_O_12_^+^, 686.3355 | 686.3350 | 0.7 |
| C_29_H_45_N_6_O_12_^+^, 669.309 | 669.312 | -5.3 |
| C_24_H_40_N_5_O_11_^+^, 574.2719 | 574.2699 | 3.4 |
| C_24_H_38_N_5_O_10_^+^, 556.2613 | 556.2621 | -1.4 |
| C_24_H_36_N_5_O_9_^+^, 538.2508 | 538.2504 | 0.7 |
| C_22_H_37_N_4_O_10_^+^, 517.2504 | 517.2488 | 3.0 |
| C_22_H_35_N_4_O_9_^+^, 499.2399 | 499.2401 | -0.4 |
| C_18_H_30_N_3_O_5_^+^, 368.2180 | 368.2178 | 0.5 |
| C_14_H_24_NO_5_^+^, 286.1649 | 286.1642 | 2.4 |

**Supplementary Table 4:** Summary of selected fragment ions of compound **2**.


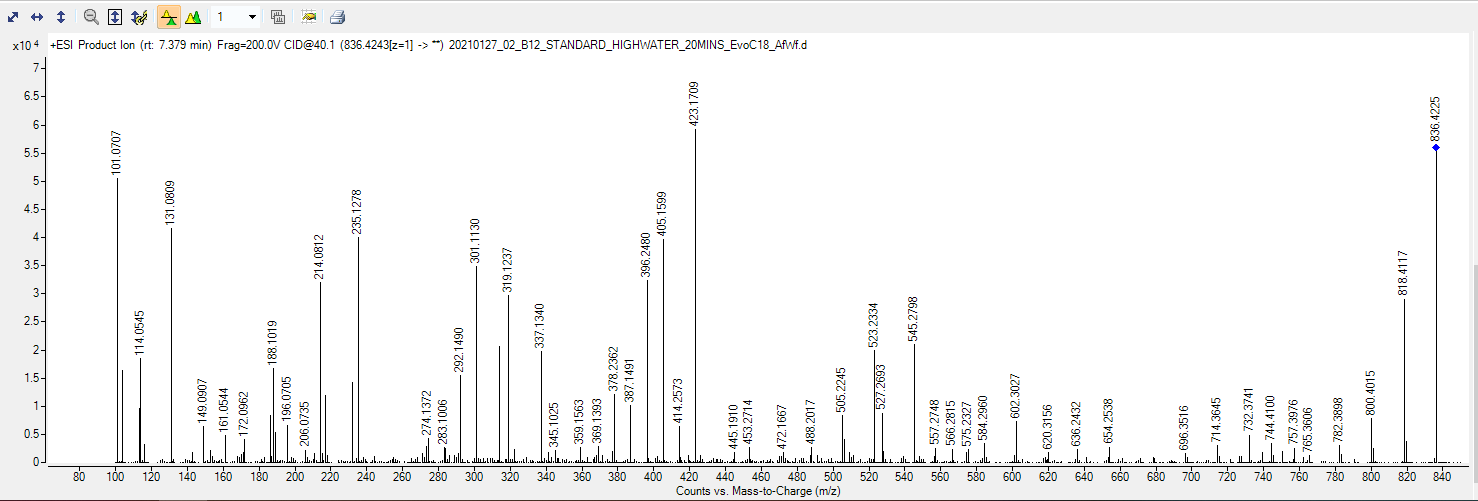


**Supplementary Figure 11:** HRMS^2^ fragment spectrum of compound **3**.

**Supplementary Figure 12:** Annotation of select fragment ions of compound 3.

| **Fragment calculated formula and *m/z*** | **Observed HRMS/MS *m/z*** | **Error (ppm)** |
| --- | --- | --- |
| C_35_H_60_N_7_O_15_^+^, 818.4142 | 818.4117 | 3.0 |
| C_35_H_58_N_7_O_14_^+^, 800.4036 | 800.4015 | 2.6 |
| C_31_H_54_N_7_O_13_^+^, 732.3774 | 732.3741 | 4.5 |
| C_31_H_52_N_7_O_12_^+^, 714.3668 | 714.3645 | 3.2 |
| C_31_H_50_N_7_O_11_^+^, 696.3563 | 696.3516 | 6.7 |
| C_26_H_44_N_5_O_11_^+^, 602.3032 | 602.3027 | 0.8 |
| C_26_H_42_N_5_O_10_^+^, 584.2926 | 584.2960 | -5.8 |
| C_26_H_40_N_5_O_9_^+^, 566.2821 | 566.2815 | 1.0 |
| C_24_H_41_N_4_O_10_^+^, 545.2817 | 545.2798 | 3.4 |
| C_24_H_39_N_4_O_9_^+^, 527.2712 | 527.2693 | 3.6 |
| C_20_H_36_N_3_O_6_^+^, 414.2599 | 414.2573 | 6.2 |
| C_20_H_34_N_3_O_5_^+^, 396.2493 | 396.2480 | 3.2 |
| C_16_H_28_NO_5_^+^, 314.1962 | 314.1955 | 2.2 |

**Supplementary Table 5:** Summary of selected fragment ions of compound **3**.


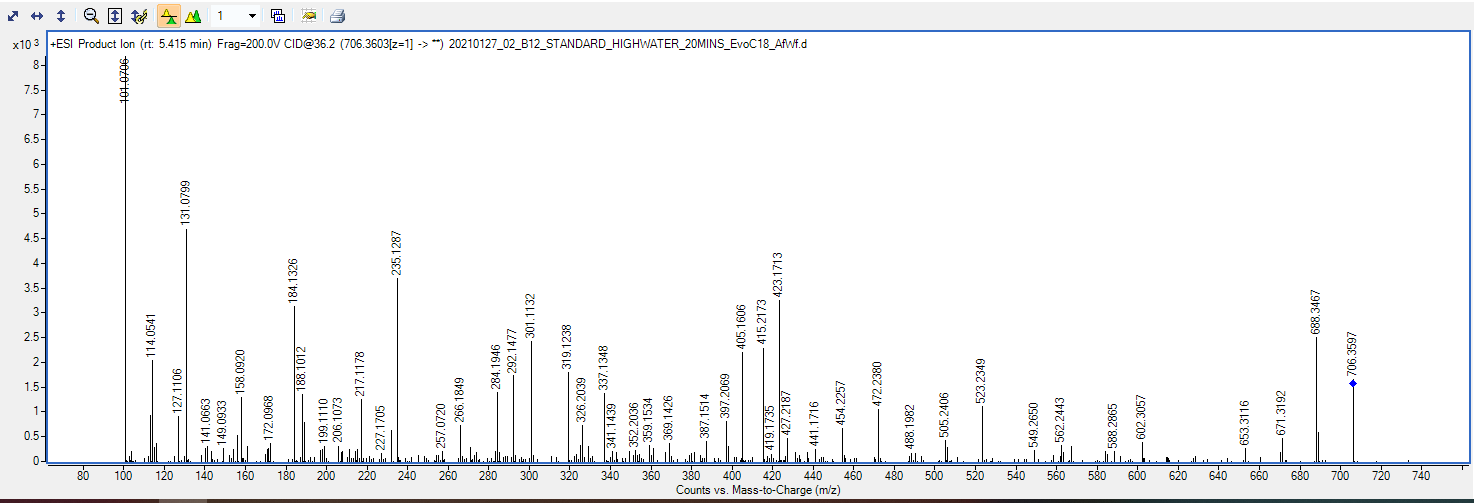


**Supplementary Figure 13:** HRMS^2^ fragment spectrum of compound **4**.

**Supplementary Figure 14:** Annotation of select fragment ions of compound **4**.

| **Fragment calculated formula and *m/z*** | **Observed HRMS/MS *m/z*** | **Error (ppm)** |
| --- | --- | --- |
| C_21_H_36_N_7_O_11_^+^, 562.2467 | 562.2443 | 4.2 |
| C_19_H_35_N_6_O_11_^+^, 523.2358 | 523.2349 | 1.7 |
| C_20_H_32_N_5_O_7_^+^, 454.2296 | 454.2257 | 8.5 |
| C_17_H_25_N_6_O_8_^+^, 441.1728 | 441.1716 | 2.7 |
| C_18_H_31_N_4_O_7_^+^, 415.2187 | 415.2173 | 3.3 |
| C_18_H_29_N_4_O_6_^+^, 397.2082 | 397.2069 | 3.2 |
| C_14_H_26_N_3_O_3_^+^, 284.1969 | 284.1946 | 8.1 |
| C_10_H_18_NO_2_^+^, 184.1332 | 184.1326 | 3.2 |
| C_6_H_12_N_3_O_2_^+^, 158.0924 | 158.0920 | 2.5 |

**Supplementary Table 6:** Summary of selected fragment ions of compound **4**.


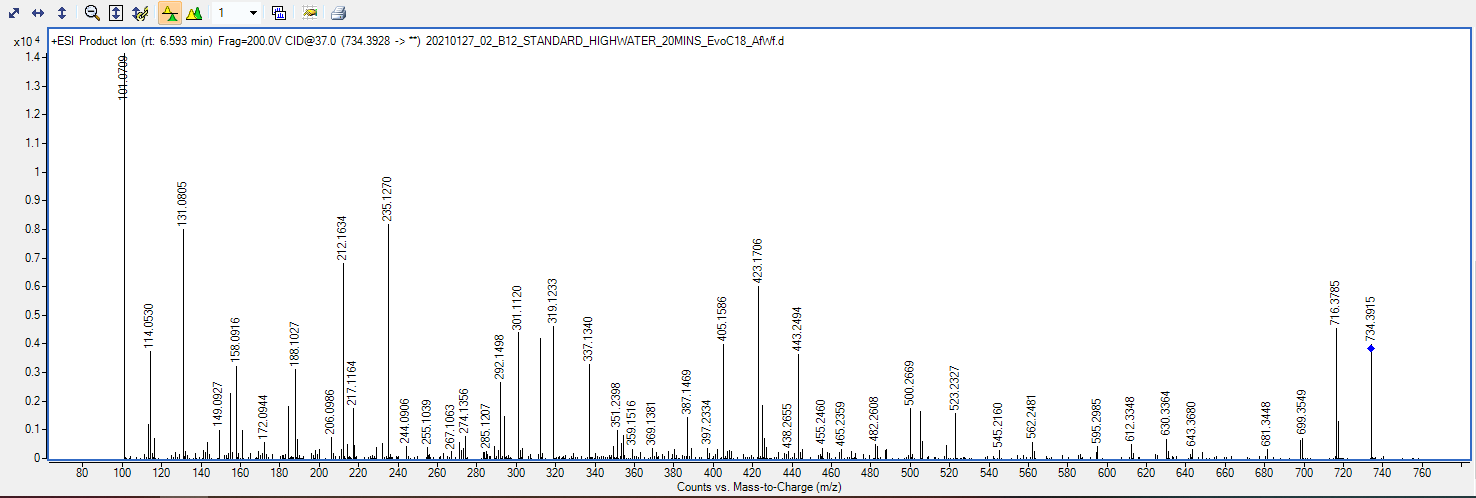


**Supplementary Figure 15:** HRMS^2^ fragment spectrum of compound **5**.

**Supplementary Figure 16:** Annotation of select fragment ions of compound **5**.

| **Fragment calculated formula and *m/z*** | **Observed HRMS/MS *m/z*** | **Error (ppm)** |
| --- | --- | --- |
| C_27_H_46_N_7_O_9_^+^, 612.3352 | 612.3348 | 0.6 |
| C_21_H_36_N_7_O_11_^+^, 562.2467 | 562.2481 | -2.4 |
| C_19_H_35_N_6_O_11_^+^, 523.2358 | 523.2327 | 5.9 |
| C_22_H_38_N_5_O_8_^+^, 500.2715 | 500.2669 | 9.0 |
| C_20_H_35_N_4_O_7_^+^, 443.2500 | 443.2494 | 1.3 |
| C_16_H_30_N_3_O_3_^+^, 312.2282 | 312.2262 | 6.4 |
| C_12_H_22_NO_2_^+^, 212.1645 | 212.1634 | 5.0 |
| C_6_H_12_N_3_O_2_^+^, 158.0924 | 158.0916 | 5.0 |

**Supplementary Table 7:** Summary of selected fragment ions of compound **5**.


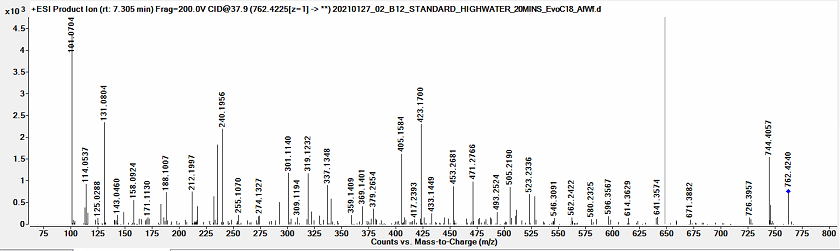


**Supplementary Figure 17:** HRMS^2^ fragment spectrum of compound **6**.

**Supplementary Figure 18:** Annotation of select fragment ions of compound **6**.

| **Fragment calculated formula and *m/z*** | **Observed HRMS/MS *m/z*** | **Error (ppm)** |
| --- | --- | --- |
| C_21_H_36_N_7_O_11_^+^, 562.2467 | 562.2422 | 8.0 |
| C_24_H_42_N_5_O_8_^+^, 528.3028 | 528.3067 | -7.3 |
| C_19_H_35_N_6_O_11_^+^, 523.2358 | 526.2336 | 4.2 |
| C_22_H_39_N_4_O_7_^+^, 471.2813 | 471.2766 | 9.9 |
| C_18_H_34_N_3_O_3_^+^, 340.2595 | 340.2582 | 3.8 |
| C_14_H_26_NO_2_^+^, 240.1958 | 240.1956 | 0.8 |
| C_6_H_12_N_3_O_2_^+^, 158.0924 | 158.0924 | 0 |

**Supplementary Table 8:** Summary of Selected Fragment Ions of compound **6**.


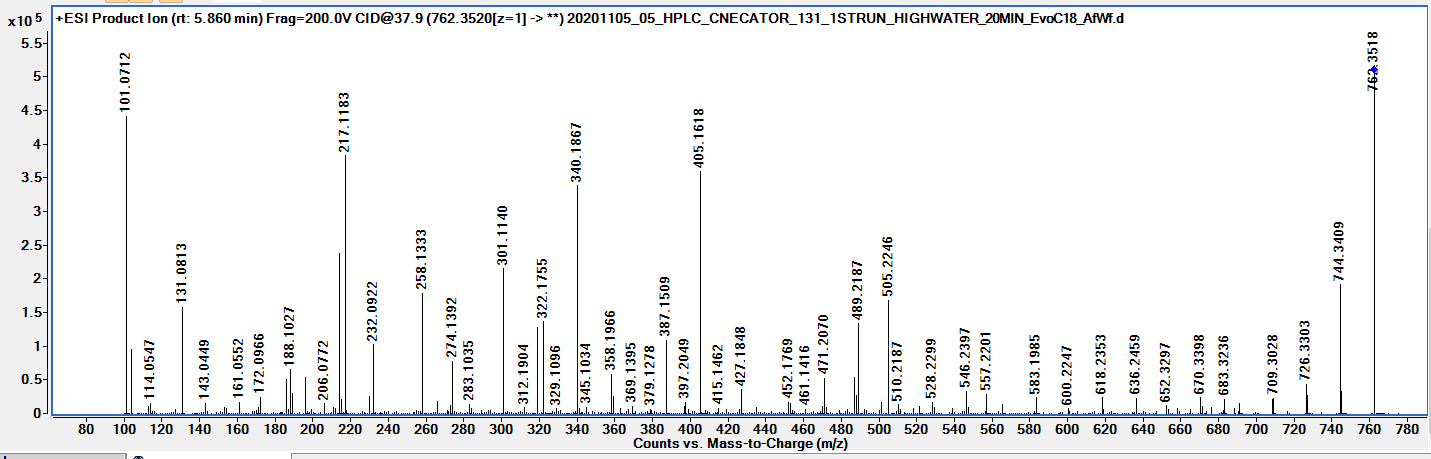


**Supplementary Figure 19:** HRMS^2^ fragment spectrum of compound **7**.

**Supplementary Figure 20:** Annotation of select fragment ions of compound **7**.

| **Fragment calculated formula and *m/z*** | **Observed HRMS/MS *m/z*** | **Error (ppm)** |
| --- | --- | --- |
| C_23_H_38_N_7_O_14_^+^, 636.2471 | 636.2459 | 1.8 |
| C_19_H_33_N_6_O_10_^+^, 505.2253 | 505.2246 | 1.4 |
| C_20_H_33_N_4_O_10_^+^, 489.2191 | 489.2187 | 0.8 |
| C_15_H_25_N_4_O_9_^+^, 405.1616 | 405.1618 | -0.5 |
| C_16_H_28_N_3_O_6_^+^, 358.1973 | 358.1966 | 2.0 |
| C_16_H_26_N_3_O_5_^+^, 340.1867 | 340.1867 | 0.0 |
| C_12_H_20_NO_5_^+^, 258.1336 | 258.1333 | 1.2 |
| C_9_H_17_N_2_O_4_^+^, 217.1183 | 217.1183 | 0 |
| C_5_H_11_N_2_O_2_^+^, 131.0815 | 131.0813 | 1.5 |

**Supplementary Table 9:** Summary of selected fragment ions of compound **7**.


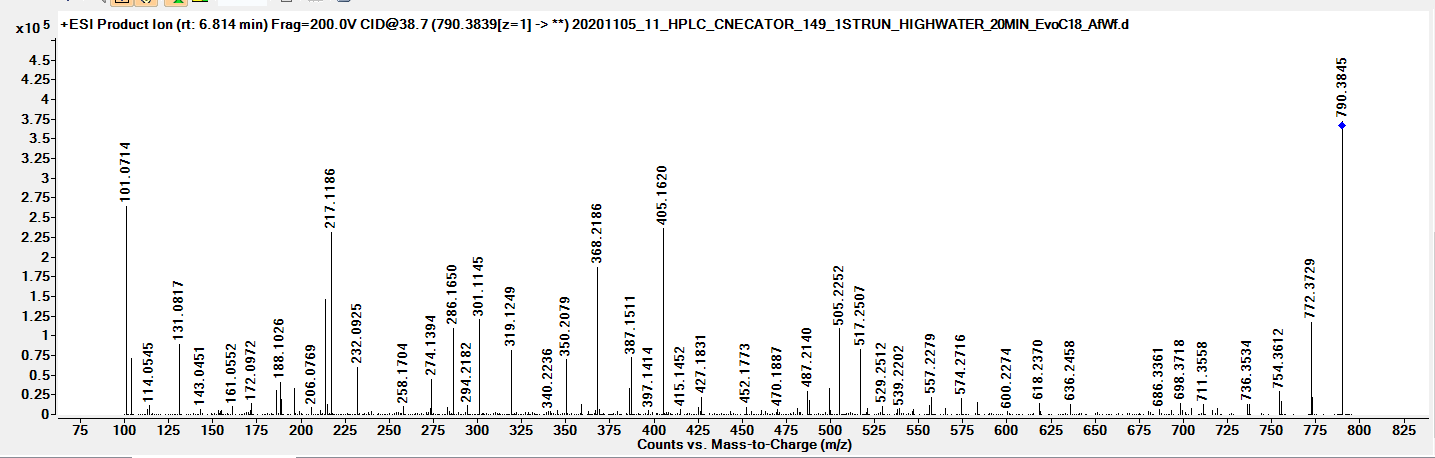


**Supplementary Figure 21:** HRMS^2^ fragment spectrum of compound **8**.

**Supplementary Figure 22:** Annotation of select fragment ions of compound **8**.

| **Fragment calculated formula and *m/z*** | **Observed HRMS/MS *m/z*** | **Error (ppm)** |
| --- | --- | --- |
| C_23_H_38_N_7_O_14_^+^, 636.2471 | 636.2458 | 2.0 |
| C_24_H_40_N_5_O_11_^+^, 574.2719 | 574.2716 | 0.5 |
| C_22_H_37_N_4_O_10_^+^, 517.2504 | 517.2507 | 0.6 |
| C_18_H_30_N_3_O_5_^+^, 368.2180 | 368.2186 | -1.6 |
| C_14_H_24_NO_5_^+^, 286.1649 | 286.1650 | 0.3 |
| C_11_H_20_N_3_O_5_^+^, 274.1397 | 274.1394 | 1.0 |
| C_9_H_17_N_2_O_4_^+^, 217.1183 | 217.1186 | -1.4 |
| C_5_H_11_N_2_O_2_^+^, 131.0815 | 131.0817 | -1.5 |
| C_4_H_9_N_2_O^+^, 101.0709 | 101.0714 | -4.9 |

**Supplementary Table 10:** Summary of selected fragment ions of compound **8**.


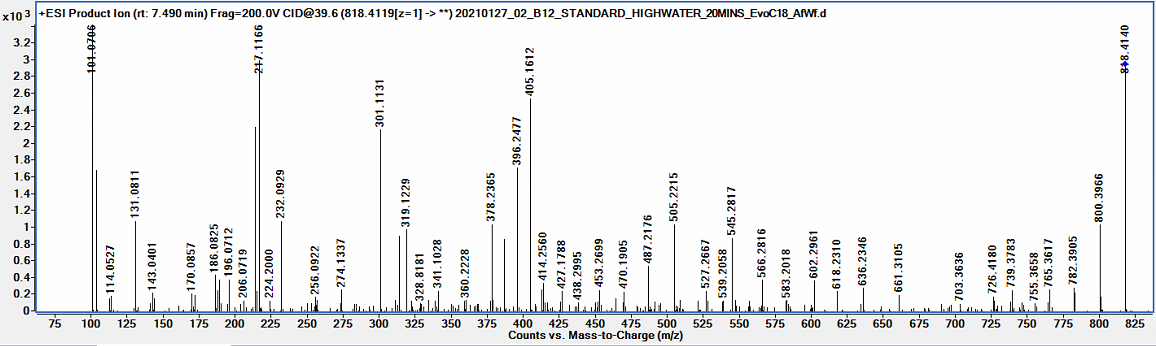


**Supplementary Figure 23:** HRMS^2^ fragment spectrum of compound **9.**

**Supplementary Figure 24:** Annotation of select fragment ions of compound **9.**

| **Fragment calculated formula and *m/z*** | **Observed HRMS/MS *m/z*** | **Error (ppm)** |
| --- | --- | --- |
| C_24_H_41_N_4_O_10_^+^, 545.2817 | 545.2817 | 0 |
| C_19_H_33_N_6_O_10_^+^, 505.2253 | 505.2215 | 7.5 |
| C_20_H_36_N_3_O_6_^+^, 414.2599 | 414.2560 | 9.4 |
| C_16_H_28_NO_5_^+^, 314.1962 | 314.195 | 2.9 |
| C_9_H_17_N_2_O_4_^+^, 217.1183 | 217.1166 | 7.8 |
| C_5_H_11_N_2_O_2_^+^, 131.0815 | 131.0811 | 3.0 |

**Supplementary Table 11:** Summary of selected fragment ions of compound **9.**


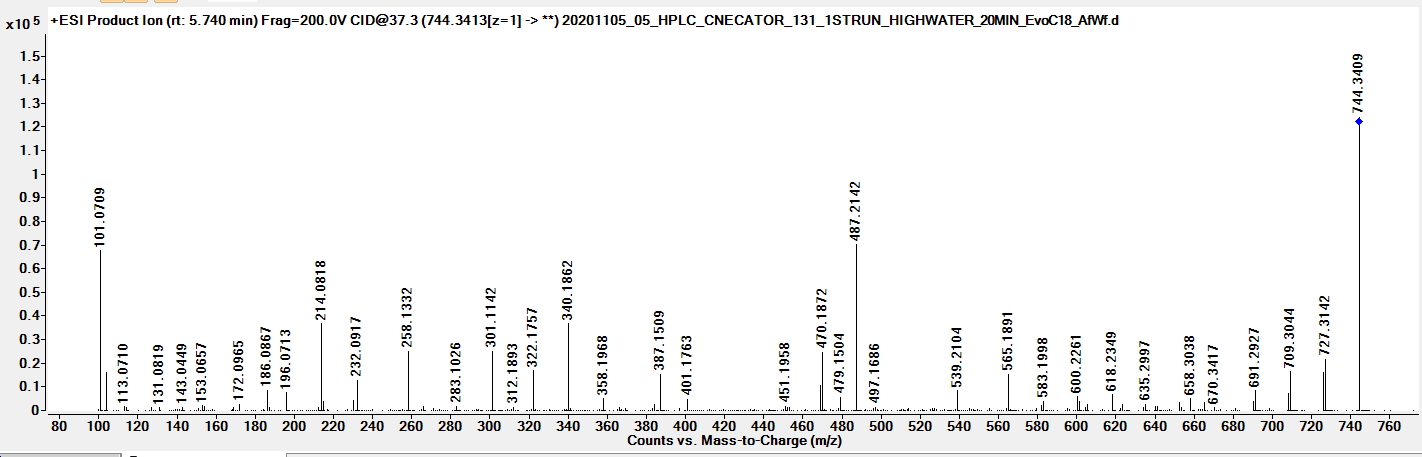


**Supplementary Figure 25:** HRMS^2^ fragment spectrum of compound **10**.

**Supplementary Figure 26:** Annotation of select fragment ions of compound **10**.

| **Fragment calculated formula and *m/z*** | **Observed HRMS/MS *m/z*** | **Error (ppm)** |
| --- | --- | --- |
| C_27_H_44_N_7_O_12_^+^, 658.3042 | 658.3038 | 0.6 |
| C_23_H_36_N_7_O_13_^+^, 618.2366 | 618.2349 | 2.7 |
| C_19_H_31_N_6_O_9_^+^, 487.2147 | 487.2142 | 1.0 |
| C_15_H_23_N_4_O_8_^+^, 387.1510 | 387.1509 | 0.3 |
| C_16_H_28_N_3_O_6_^+^, 358.1973 | 358.1968 | 1.4 |
| C_11_H_17_N_4_O_6_^+^, 301.1143 | 301.1142 | 0.3 |
| C_12_H_20_NO_5_^+^, 258.1336 | 258.1332 | 1.5 |
| C_4_H_9_N_2_O^+^, 101.0709 | 101.0709 | 0 |

**Supplementary Table 12:** Summary of selected fragment ions of compound **10**.


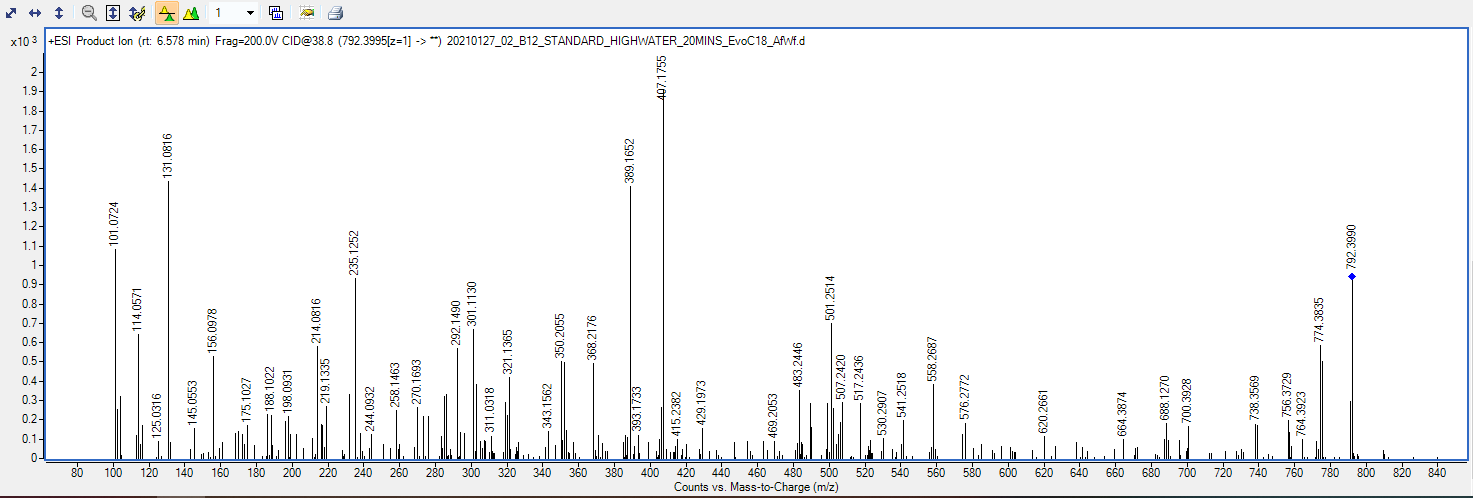


**Supplementary Figure 27:** MS^2^ fragment spectrum of compound **11**.

**Supplementary Figure 28:** Annotation of select fragment ions of compound **11**.

| **Fragment calculated formula and *m/z*** | **Observed HRMS/MS *m/z*** | **Error (ppm)** |
| --- | --- | --- |
| C_22_H_37_N_4_O_9_^+^, 501.2555 | 501.2514 | 8.1 |
| C_15_H_27_N_4_O_9_^+^, 407.1773 | 407.1755 | 4.4 |
| C_15_H_25_N_4_O_8_^+^, 389.1667 | 389.1652 | 3.8 |
| C_18_H_30_N_3_O_5_^+^, 368.2180 | 368.2176 | 1.0 |
| C_11_H_22_N_3_O_6_^+^, 292.1503 | 292.1490 | 4.4 |
| C_14_H_24_NO_5_^+^, 286.1649 | 286.1618 | 10. |
| C_8_H_12_N_3_O_4_^+^, 214.0822 | 214.0816 | 2.8 |

**Supplementary Table 13:** Summary of selected fragment ions of compound **11**.


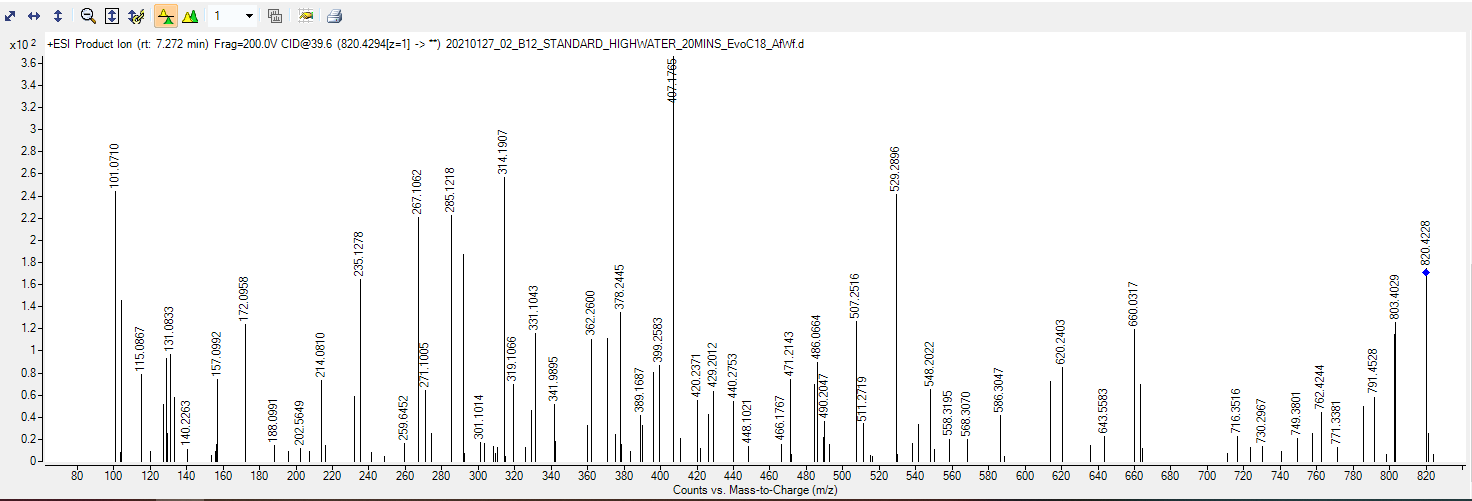


**Supplementary Figure 29:** HRMS^2^ fragment spectrum of compound **12**.

**Supplementary Figure 30:** Annotation of select fragment ions of compound **12**.

| **Fragment calculated formula and *m/z*** | **Observed HRMS/MS *m/z*** | **Error (ppm)** |
| --- | --- | --- |
| C_24_H_41_N_4_O_9_^+^, 529.2868 | 529.2896 | -5.2 |
| C_15_H_27_N_4_O_9_^+^, 407.1773 | 407.1765 | 2.0 |
| C_20_H_34_N_3_O_5_^+^, 396.2493 | 396.2477 | 4.0 |
| C_11_H_22_N_3_O_6_^+^, 292.1503 | 292.1491 | 4.1 |
| C_9_H_19_N_2_O_5_^+^, 235.1288 | 235.1278 | 4.2 |
| C_8_H_12_N_3_O_4_^+^, 214.0822 | 214.0810 | 5.6 |
| C_4_H_9_N_2_O^+^, 101.0709 | 101.0710 | -1.0 |

**Supplementary Table 14:** Summary of selected fragment ions of compound **12**.


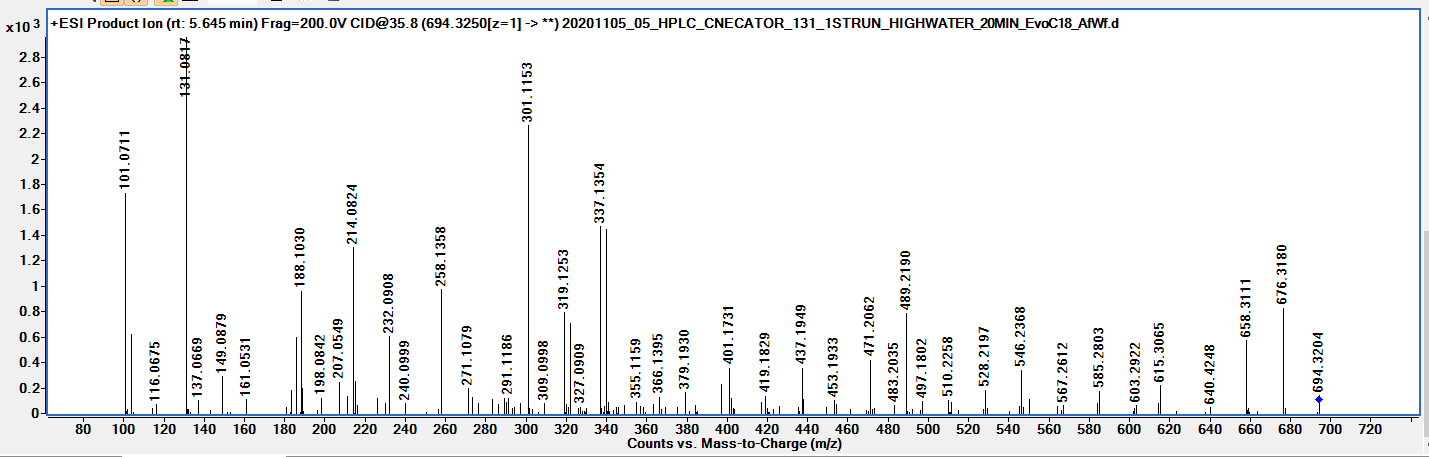


**Supplementary Figure 31:** HRMS^2^ fragment spectrum of compound **13**.

**Supplementary Figure 32:** Annotation of select fragment ions of compound **13**.

| **Fragment calculated formula and *m/z*** | **Observed HRMS/MS *m/z*** | **Error (ppm)** |
| --- | --- | --- |
| C_22_H_36_N_5_O_11_^+^, 546.2406 | 546.2368 | 6.9 |
| C_20_H_33_N_4_O_10_^+^, 489.2191 | 489.2190 | 0.2 |
| C_11_H_21_N_4_O_8_^+^, 337.1354 | 337.1354 | 0 |
| C_11_H_19_N_4_O_7_^+^, 319.1248 | 319.1253 | -1.5 |
| C_11_H_17_N_4_O_6_^+^, 301.1143 | 301.1153 | -3.3 |
| C_12_H_20_NO_5_^+^, 258.1336 | 258.1358 | 8.5 |
| C_8_H_14_N_3_O_5_^+^, 232.0928 | 232.0908 | 8.6 |
| C_8_H_12_N_3_O_4_^+^, 214.0822 | 214.0824 | -0.9 |
| C_5_H_11_N_2_O_2_^+^, 131.0815 | 131.0817 | -1.5 |

**Supplementary Table 15:** Summary of selected fragment ions of compound **13**.


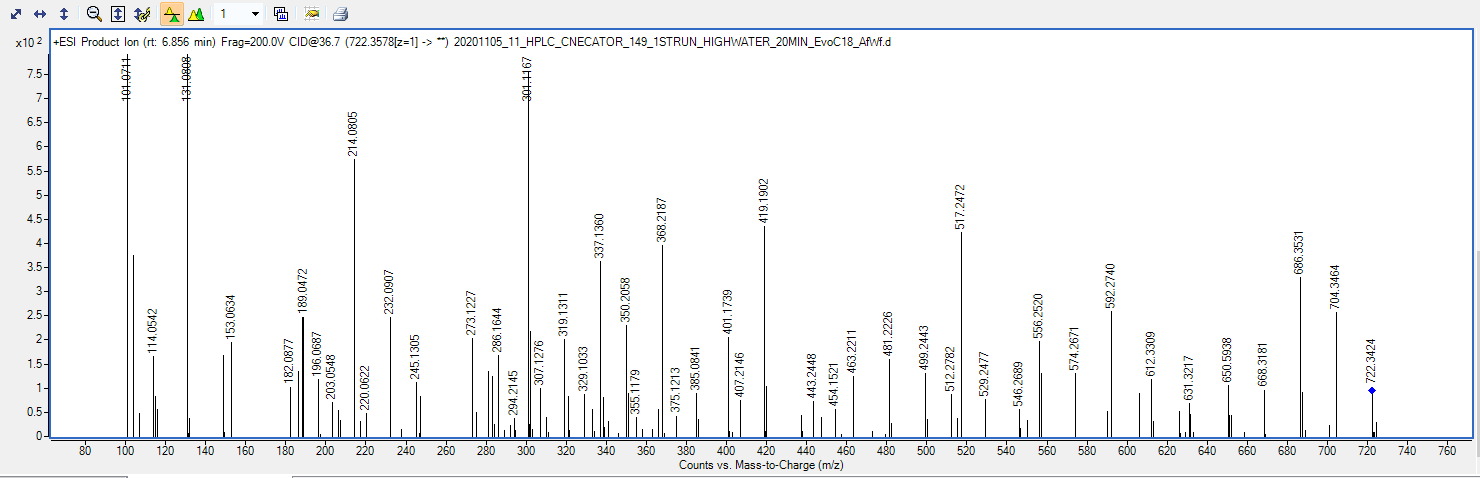


**Supplementary Figure 33:** HRMS^2^ fragment spectrum of compound **14**.

**Supplementary Figure 34:** Annotation of select fragment ions of compound **14**.

| **Fragment calculated formula and *m/z*** | **Observed HRMS/MS *m/z*** | **Error (ppm)** |
| --- | --- | --- |
| C_24_H_40_N_5_O_11_^+^, 574.2719 | 574.2671 | 8.3 |
| C_22_H_37_N_4_O_10_^+^, 517.2504 | 517.2472 | 6.2 |
| C_11_H_21_N_4_O_8_^+^, 337.1354 | 337.1360 | -1.8 |
| C_11_H_17_N_4_O_6_^+^, 301.1143 | 301.1167 | -7.9 |
| C_14_H_24_NO_5_^+^, 286.1649 | 286.1644 | 1.7 |
| C_8_H_14_N_3_O_5_^+^, 232.0928 | 232.0907 | 9.0 |
| C_8_H_12_N_3_O_4_^+^, 214.0822 | 214.0805 | 7.9 |
| C_5_H_11_N_2_O_2_^+^, 131.0815 | 131.0808 | 5.3 |
| C_4_H_9_N_2_O^+^, 101.0709 | 101.0711 | -1.9 |

**Supplementary Table 16:** Summary of selected fragment ions of compound **14**.


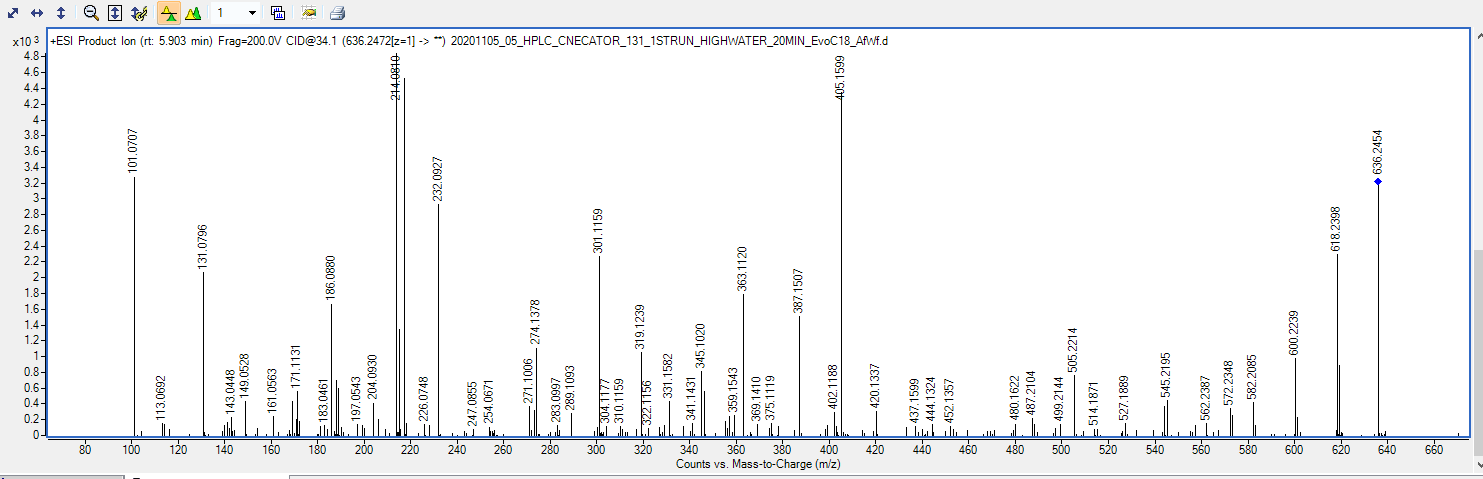


**Supplementary Figure 35:** HRMS^2^ fragment spectrum of compound **15**.

**Supplementary Figure 36:** Annotation of select fragment ions of compound **15**.

| **Fragment calculated formula and *m/z*** | **Observed HRMS/MS *m/z*** | **Error (ppm)** |
| --- | --- | --- |
| C_23_H_36_N_7_O_13_^+^, 618.2366 | 618.2398 | -5.1 |
| C_19_H_33_N_6_O_10_^+^, 505.2253 | 505.2214 | 7.7 |
| C_15_H_25_N_4_O_9_^+^, 405.1616 | 405.1599 | 4.1 |
| C_15_H_23_N_4_O_8_^+^, 387.1510 | 387.1507 | 0.8 |
| C_11_H_19_N_4_O_7_^+^, 319.124 | 319.1239 | 2.8 |
| C_11_H_17_N_4_O_6_^+^, 301.1143 | 301.1159 | -5.3 |
| C_11_H_20_N_3_O_5_^+^, 274.1397 | 274.1378 | 6.9 |
| C_8_H_14_N_3_O_5_^+^, 232.0928 | 232.0927 | 0.4 |
| C_8_H_12_N_3_O_4_^+^, 214.0822 | 214.0810 | 5.6 |
| C_4_H_9_N_2_O^+^, 101.0709 | 101.0707 | 1.9 |

**Supplementary Table 17:** Summary of selected fragment ions of compound **15**.


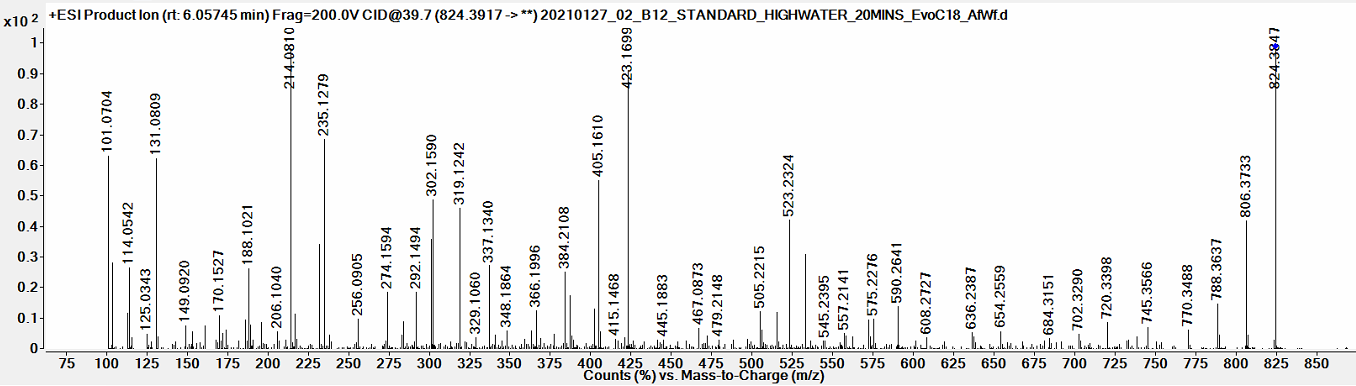


**Supplementary Figure 37:** HRMS^2^ fragment spectrum of compound **16**.

**Supplementary Figure 38: Annotation of select fragment ions of compound 16.**

| **Fragment calculated formula and *m/z*** | **Observed HRMS/MS *m/z*** | **Error (ppm)** |
| --- | --- | --- |
| C_29_H_50_N_7_O_14_^+^, 720.3410 | 720.3398 | 1.6 |
| C_24_H_40_N_5_O_12_^+^, 590.2668 | 590.2641 | 4.7 |
| C_22_H_37_N_4_O_11_^+^, 533.2448 | 533.2445 | 0.5 |
| C_19_H_35_N_6_O_11_^+^, 523.2358 | 523.2324 | 6.4 |
| C_22_H_35_N_4_O_10_^+^, 515.2348 | 515.2305 | 8.3 |
| C_19_H_33_N_6_O_10_^+^, 505.2253 | 505.2215 | 7.5 |
| C_15_H_27_N_4_O_10_^+^, 423.1722 | 423.1699 | 5.4 |
| C_15_H_25_N_4_O_9_^+^, 405.1616 | 405.1610 | 1.4 |
| C_18_H_30_N_3_O_6_^+^, 384.2124 | 384.2108 | 4.1 |
| C_14_H_24_NO_6_^+^, 302.1598 | 302.1590 | 2.6 |
| C_11_H_22_N_3_O_6_^+^, 292.1503 | 292.1494 | 3.0 |
| C_9_H_19_N_2_O_5_^+^, 235.1288 | 235.1279 | 3.8 |

**Supplementary Table 18: Summary of selected fragment ions of compound 16.**


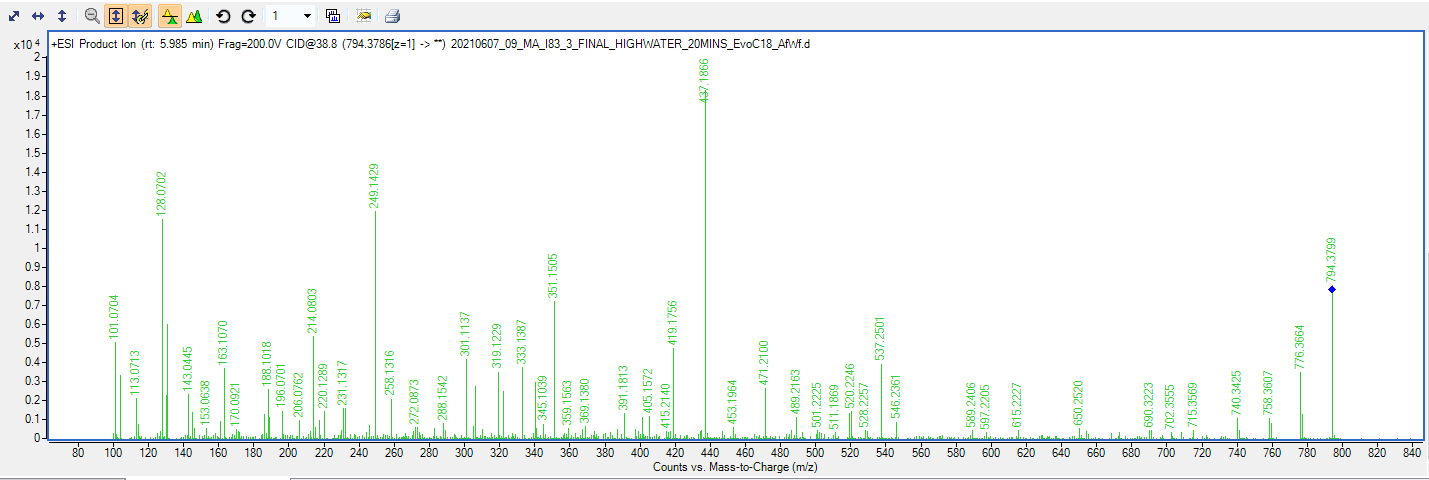


**Supplementary Figure 39:** HRMS^2^ fragment spectrum of compound **17**.

**Supplementary Figure 40:** Annotation of select fragment ions of compound **17**.

| **Fragment calculated formula and *m/z*** | **Observed HRMS/MS *m/z*** | **Error (ppm)** |
| --- | --- | --- |
| C_32_H_54_N_7_O_15_^+^, 776.3672 | 776.3664 | 1.0 |
| C_32_H_52_N_7_O_14_^+^, 758.3567 | 758.3607 | -5.2 |
| C_32_H_50_N_7_O_13_^+^, 740.3461 | 740.3425 | 4.8 |
| C_24_H_35_N_6_O_13_^+^, 615.2257 | 615.2227 | 4.8 |
| C_19_H_35_N_6_O_11_^+^, 523.2358 | 523.2350 | 1.5 |
| C_22_H_36_N_5_O_11_^+^, 546.2406 | 546.2361 | 8.2 |
| C_20_H_37_N_6_O_11_^+^, 537.2515 | 537.2501 | 2.6 |
| C_22_H_34_N_5_O_10_^+^, 528.2300 | 528.2257 | 8.1 |
| C_20_H_33_N_4_O_10_^+^, 489.2191 | 489.2163 | 5.7 |
| C_20_H_31_N_4_O_9_^+^, 471.2086 | 471.2100 | -2.9 |
| C_20_H_29_N_4_O_8_^+^, 453.1980 | 453.1964 | 3.5 |
| C_16_H_29_N_4_O_10_^+^, 437.1878 | 437.1866 | 2.7 |
| C_16_H_27_N_4_O_9_^+^, 419.1773 | 419.1756 | 4.0 |
| C_12_H_22_N_3_O_5_^+^, 288.1554 | 288.1542 | 4.1 |
| C_12_H_20_NO_5_^+^, 258.1336 | 258.1316 | 7.7 |
| C_10_H_21_N_2_O_5_^+^, 249.1445 | 249.1429 | 6.4 |
| C_6_H_10_NO_2_^+^, 128.0706 | 128.0702 | 3.1 |
| C_4_H_9_N_2_O^+^, 101.0709 | 101.0704 | 4.9 |

**Supplementary Table 19:** Summary of selected fragment ions of compound 17.

## Marfey’s analysis of compound 1.

**
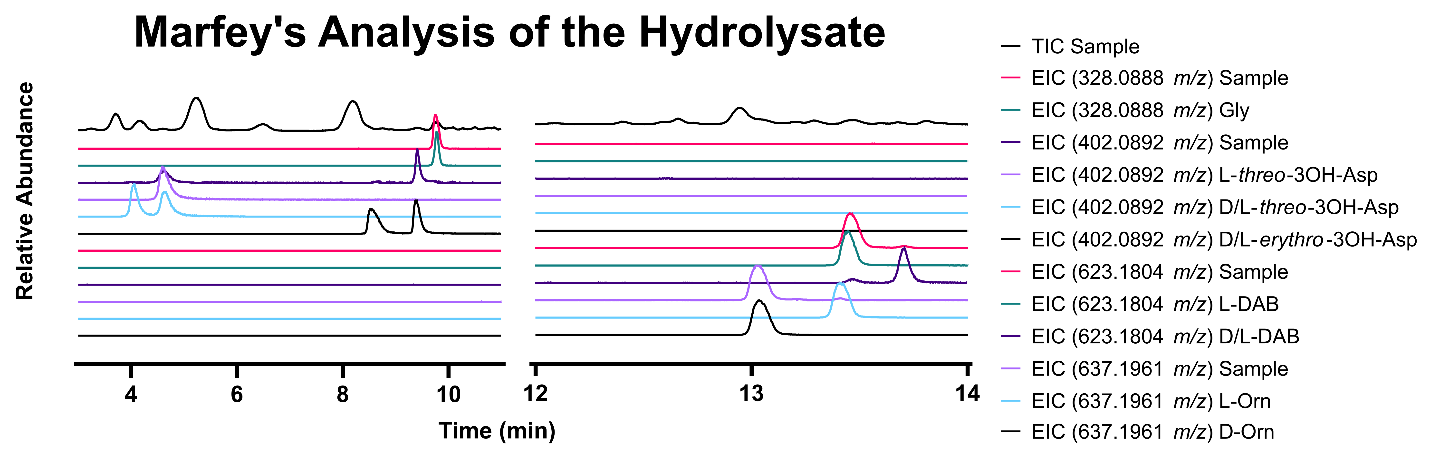
**

**Supplementary Figure 41:** LCMS chromatograms from the Marfey’s analysis. The LCMS Total Ion Chromatogram (TIC) for the hydrolysate for **1** and the Extracted Ion Chromatogram for the Marfey’s labeled amino acids in both the sample and the glycine, β-hydroxyaspartic acid, 2,4-diaminobutyrate, and ornithine standards.

## HRMS fragment spectra, fragment annotations, and summary tables from the UV exposure experiments.


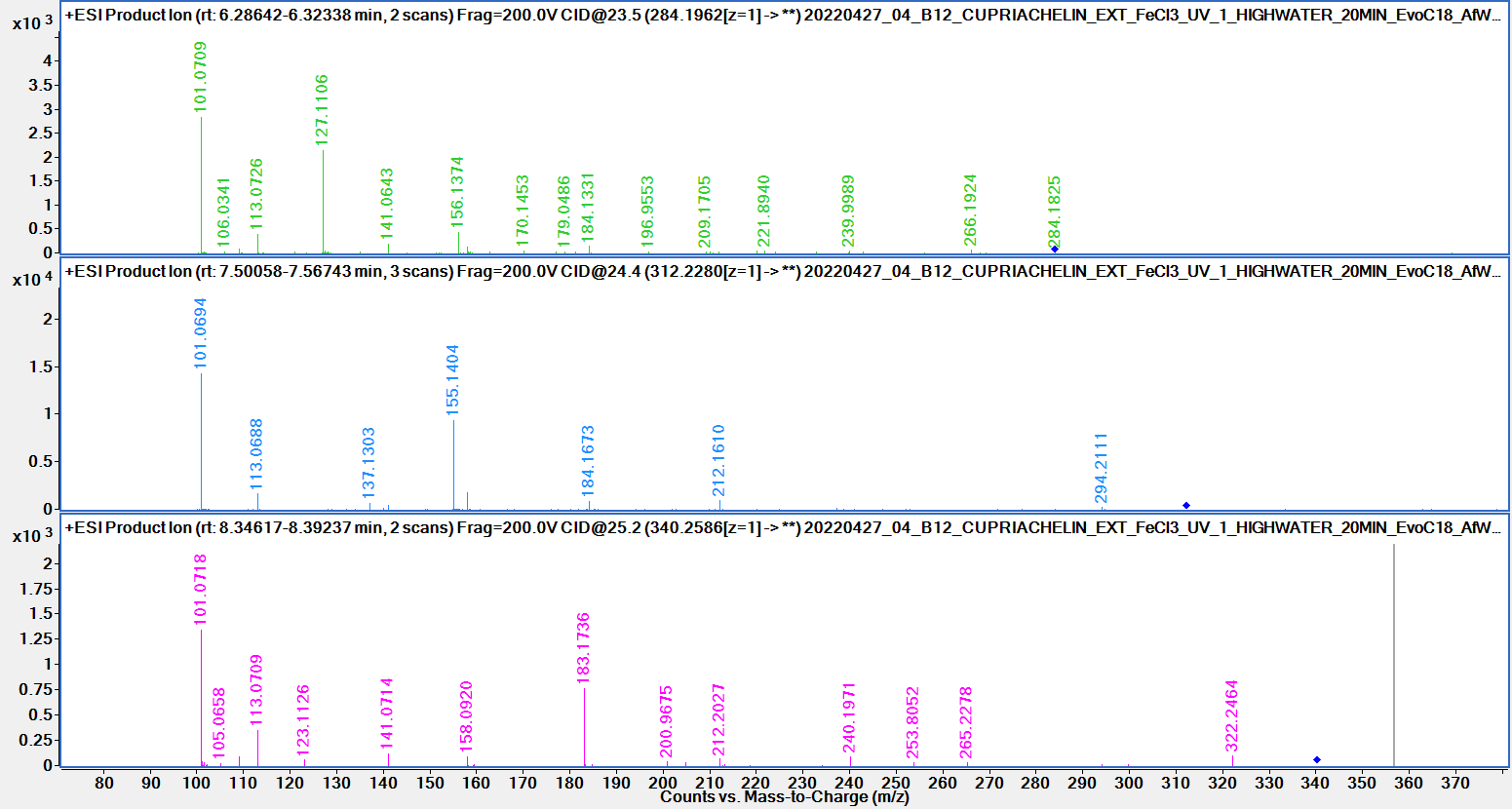


**Supplementary Figure 42:** HRMS^2^ fragment spectra of compounds **18-20** from the initial UV exposure experiment. Spectra for **18**, **19**, and **20** in descending order with **18** on the top.

**Supplementary Figure 43:** Annotation of compounds **18-20** from the initial UV exposure experiment.

1. **^13^C-labeled Isotopologue.**

**
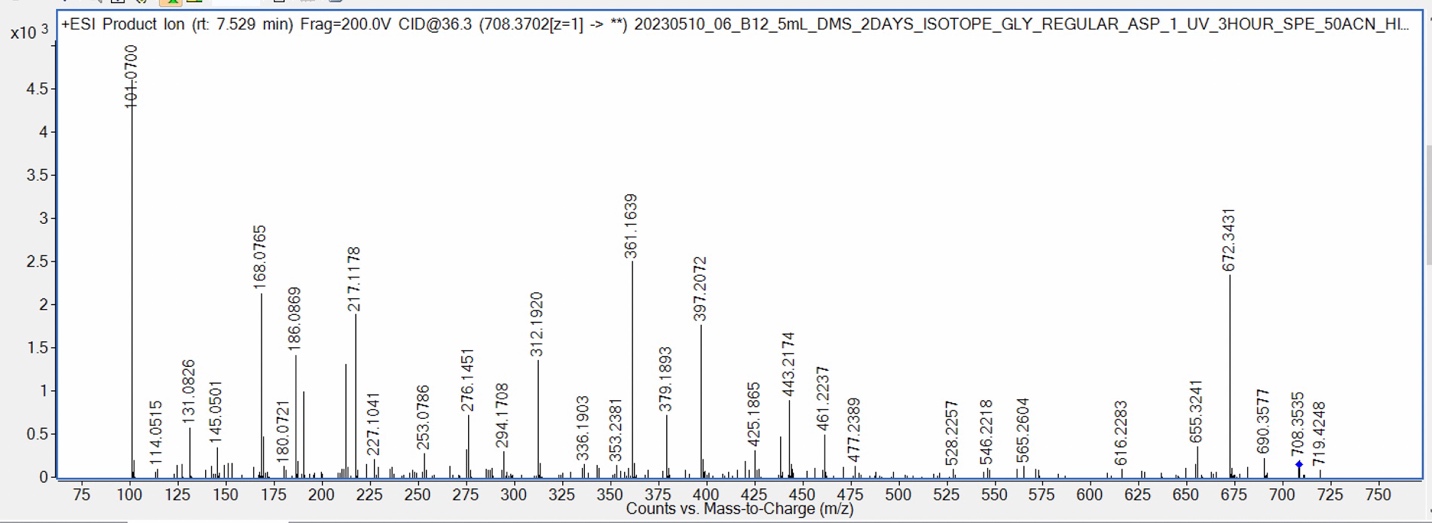
**

1. **^15^N-labeled Isotopologue.**

**
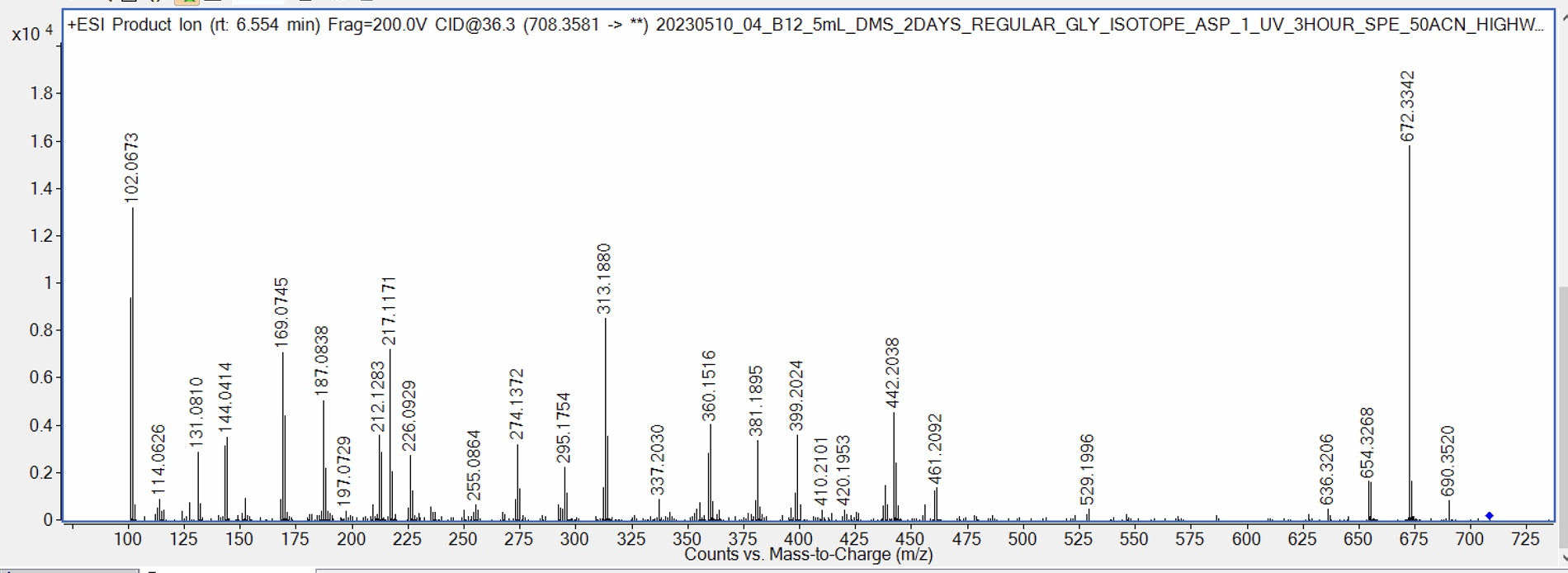
**

**Supplementary Figure 44:** HRMS^2^ Fragment Spectra of Labeled Isotopologues of 4. **A.)** Spectrum of compound **4** from culture growth with (1,2)^13^C-labeled glycine. **B.)** Spectrum of compound **4** from culture grown with ^15^N-labeled L-aspartic acid.

**Supplementary Figure 45:** Annotation of labeled isotopologues of compound **4**.

| **Compound** | **Fragment calculated formula and *m/z*** | **Observed HRMS/MS *m/z*** | **Error (ppm)** |
| --- | --- | --- | --- |
| **^13^C-labeled-4** | C_27_^13^C_2_H_52_N_7_O_13_^+^, 708.3685 | 708.3702 | 2.4 |
|  | C_19_^13^C_2_H_32_N_7_O_9_^+^, 528.2323 | 528.2257 | -12.5 |
|  | C_13_^13^C_2_H_27_N_4_O_10_^+^, 425.1789 | 425.1865 | 17.9 |
|  | C_18_H_29_N_4_O_6_^+^, 397.2082 | 397.2072 | -2.5 |
|  | C_9_^13^C_2_H_20_N_3_O_5_^+^, 276.1465 | 276.1451 | -5.1 |
|  | C_5_^13^C_2_H_14_N_3_O_3_^+^, 190.1097 | 190.1096 | -0.5 |
| **^15^N-labeled-4** | C_29_H_52_N_5_^15^N_2_O_13_^+^, 708.3558 | 708.3581 | 3.2 |
|  | C_20_H_32_N_3_^15^N_2_O_7_^+^, 456.2237 | 456.2288 | 11.2 |
|  | C_18_H_29_N_2_^15^N_2_O_6_^+^, 399.2022 | 399.2024 | 0.5 |
|  | C_11_H_22_N_3_O_6_^+^, 292.1503 | 292.1482 | -7.1 |
|  | C_11_H_20_N_3_O_5_^+^, 274.1397 | 274.1372 | -9.1 |

**Supplementary Table 20:** Summary of selected fragment ions of Isotopologues of compound **4**.

## Annotation of iron adducts of compounds 1-3.


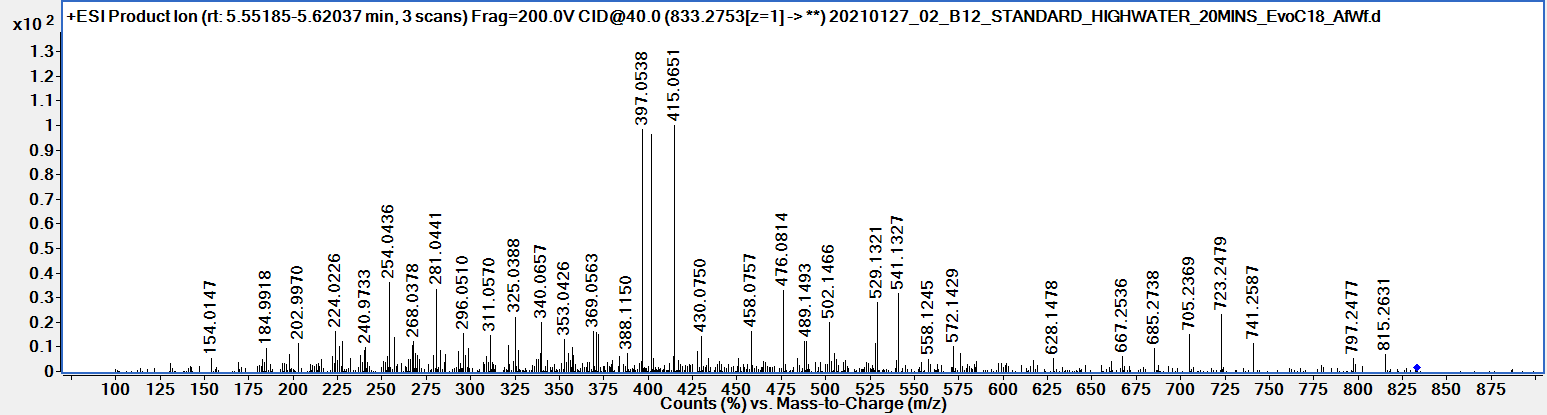

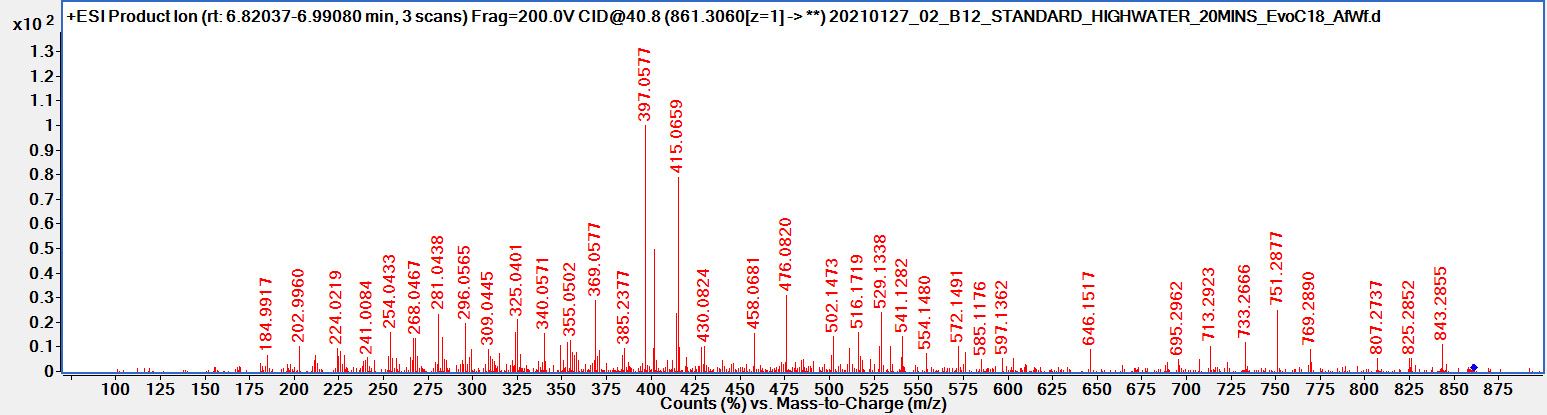

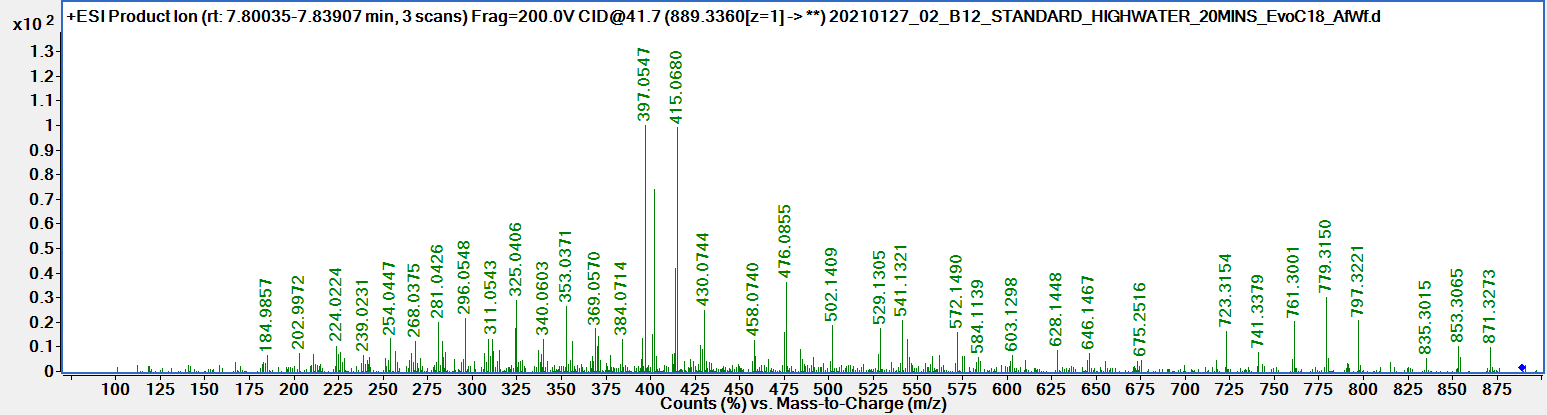


**Supplementary Figure 46:** HRMS^2^ fragment spectra of compounds **1-3** as [M-2H+Fe]^+^ adducts. Spectra for compound **1-3** in descending order, with compound **1** at the top.

**Supplementary Figure 47:** Annotation of fragments of compounds 1-3 as [M-2H+Fe]^+^ adducts.

| **Fragment ions of [M-2H+Fe]^+^ Adducts** | **Fragment calculated formula and *m/z*** | **Observed HRMS/MS *m/z*** | **Error (ppm)** |
| --- | --- | --- | --- |
| **1** | C_31_H_49_FeN_7_O_15_^+^, 815.2631 | 815.2631 | 0 |
|  | C_31_H_47_FeN_7_O_14_^+^, 797.2525 | 797.2477 | -6 |
|  | C_18_H_27_FeN_7_O_9_^+^, 541.1214 | 541.1327 | 20.9 |
|  | C_15_H_24_FeN_4_O_10_^+^, 476.0836 | 476.0814 | -4.6 |
|  | C_15_H_22_FeN_4_O_9_^+^, 458.0731 | 458.0757 | 5.7 |
| **2** | C_33_H_53_FeN_7_O_15_^+^, 843.2944 | 843.2855 | -10.6 |
|  | C_33_H_51_FeN_7_O_14_^+^, 825.2838 | 825.2852 | 1.7 |
|  | C_33_H_49_FeN_7_O_13_^+^, 807.2732 | 807.2737 | 0.6 |
|  | C_19_H_27_FeN_7_O_11_^+^, 585.1112 | 585.1176 | 10.9 |
|  | C_18_H_27_FeN_7_O_9_^+^, 541.1214 | 541.1282 | 12.6 |
|  | C_15_H_24_FeN_4_O_10_^+^, 476.0836 | 476.082 | -3.4 |
|  | C_15_H_22_FeN_4_O_9_^+^, 458.0731 | 458.0681 | -10.9 |
| **3** | C_35_H_57_FeN_7_O_15_^+^, 871.3257 | 871.3273 | 1.8 |
|  | C_35_H_55_FeN_7_O_14_^+^, 853.3151 | 853.3065 | -10.1 |
|  | C_35_H_53_FeN_7_O_13_^+^, 835.3045 | 835.3015 | -3.6 |
|  | C_18_H_27_FeN_7_O_9_^+^, 541.1214 | 541.1321 | 19.8 |
|  | C_15_H_24_FeN_4_O_10_^+^, 476.0836 | 476.0855 | 4.0 |
|  | C_15_H_22_FeN_4_O_9_^+^, 458.0731 | 458.074 | 2.0 |

**Supplementary Table 21:** Summary of selected fragment ions of compounds **1-3** as [M-2H+Fe]^+^ adducts.

## NMR shift comparison of cupriachelin A and compound 1.

| Residue | Position | δ_H_, Cupriachelin A^a^ | δ_H_, Compound **1**^b^ | Difference |
| --- | --- | --- | --- | --- |
| Octanoic acid | C-2 | 2.36 | 2.34 | 0.02 |
|  | C-3 | 1.60 | 1.59 | 0.01 |
|  | C-4 | 1.26 | 1.29 | -0.03 |
|  | C-5 | 1.26 | 1.29 | -0.03 |
|  | C-6 | 1.26 | 1.26 | 0.00 |
|  | C-7 | 1.27^c^ | 1.27 | 0.00 |
|  | C-8 | 0.85 ^c^ | 0.85 | 0.00 |
| L-*erythro*-*β*-OH-Asp-1 | C-2 | 4.81 | 4.61 | 0.20 |
|  | C-3 | 4.50 | 4.33 | 0.17 |
| L-Dab | C-2 | 4.55 | 4.54 | 0.01 |
|  | C-3_a_ | 2.26 | 2.77 | -0.51 |
|  | C-3_b_ | 2.10 | 2.09 | 0.02 |
|  | C-4 | 3.08 | 3.09 | 0.00 |
| L-*threo*-*β*-OH-Asp-2 | C-2 | 4.98 | 4.86 | 0.12 |
|  | C-3 | 4.82 | 4.58 | 0.24 |
| Gly | C-2 | 4.01 | 3.98 | 0.03 |
| D-N^δ^-OH-Orn | C-2 | 4.32 | 4.27 | 0.05 |
|  | C-3_a_ | 1.83 | 1.85 | -0.02 |
|  | C-3_b_ | 1.73 | 1.71 | 0.02 |
|  | C-4 | 1.73 | 1.67 | 0.06 |
|  | C-5 | 3.67 | 3.66 | 0.01 |
| L-Hbu | C-2_a_ | 2.78 | 2.78 | 0.00 |
|  | C-2_b_ | 2.61 | 2.61 | 0.00 |
|  | C-3 | 4.23 | 4.22 | 0.01 |
|  | C-4 | 1.23 | 1.23 | 0.00 |
|  |  |  | Mean Difference: | **0.01** |

**Supplementary Table 22:** Shift comparison between the literature report of cupriachelin A and compound 1. (^a^) Shifts as reported in Kreutzer, M.; et al. *J. Am. Chem. Soc.* 2012, *134*, 5415-5422. (^b^) Center-point of multiplets used in the calculation. (^c^) Position C-9 and C-10 in Cupriachelin A as that molecule contains decanoic acid not octanoic acid.
